# Stable Platform for Mevalonate Bioproduction from CO_2_

**DOI:** 10.1021/acssuschemeng.4c03561

**Published:** 2024-08-26

**Authors:** Marco Garavaglia, Callum McGregor, Rajesh Reddy Bommareddy, Victor Irorere, Christian Arenas, Alberto Robazza, Nigel Peter Minton, Katalin Kovacs

**Affiliations:** † BBSRC/EPSRC Synthetic Biology Research Centre (SBRC), Biodiscovery Institute, School of Life Sciences, The University of Nottingham, Nottingham NG7 2RD, U.K.; ‡ Better Dairy Limited, Unit J/K Bagel Factory, 24 White Post Lane, London E9 5SZ, U.K.; § Hub for Biotechnology in the Built Environment, Department of Applied Sciences, Faculty of Health and Life Sciences, Northumbria University, Ellison Building, Newcastle upon Tyne NE1 8ST, U.K.; ∥ DSM-Firmenich, 250 Plainsboro Road, Plainsboro, New Jersey 08536, United States; ⊥ Karlsruhe Institute of Technology (KIT), PO Box 6980, Karlsruhe 76049, Germany; # School of Pharmacy, University Park, The University of Nottingham, Nottingham NG7 2RD, U.K.

**Keywords:** *Cupriavidus necator* H16, plasmid addiction systems, mevalonate production, PHB, autotrophic gas fermentation, CO_2_ utilization

## Abstract

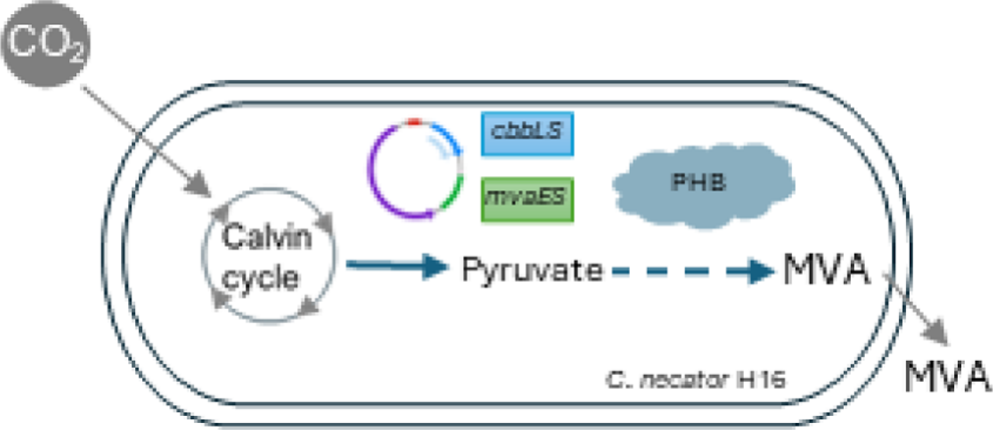

Stable production of value-added products using a microbial chassis is pivotal for determining the industrial suitability of the engineered biocatalyst. Microbial cells often lose the multicopy expression plasmids during long-term cultivations. Owing to the advantages related to titers, yields, and productivities when using a multicopy expression system compared with genomic integrations, plasmid stability is essential for industrially relevant biobased processes. *Cupriavidus necator* H16, a facultative chemolithoautotrophic bacterium, has been successfully engineered to convert inorganic carbon obtained from CO_2_ fixation into value-added products. The application of this unique capability in the biotech industry has been hindered by *C*. *necator* H16 inability to stably maintain multicopy plasmids. In this study, we designed and tested plasmid addiction systems based on the complementation of essential genes. Among these, implementation of a plasmid addiction tool based on the complementation of mutants lacking RubisCO, which is essential for CO_2_ fixation, successfully stabilized a multicopy plasmid. Expressing the mevalonate pathway operon (MvaES) using this addiction system resulted in the production of ∼10 g/L mevalonate with carbon yields of ∼25%. The mevalonate titers and yields obtained here using CO_2_ are the highest achieved to date for the production of C6 compounds from C1 feedstocks.

## Abstract 2

Bioproduction of mevalonate (MVA) from CO_2_ by *Cupriavidus necator* H16, using a plasmid addition system based on the complementation of the RuBisCO subunit (*cbbLS*) and the heterologous expression of the mevalonate pathway genes (*mvaES*).

## 1 Introduction

Serious global issues arising from climate change include extreme weather events, increasing disparity in food supply between developed and developing countries, and increased pest range due to altered weather patterns.^1,2^ Considering the global impact of major and potentially irreversible climate change, it is critical to implement measures to reduce atmospheric levels of potent greenhouse gas (GHG) CO_2_. This can be achieved either by decreasing CO_2_ emissions or by removing the CO_2_ already present in the atmosphere.

Carbon capture techniques can be employed to sequester CO_2_ from the atmosphere, reducing its effect on global warming. Moreover, the need for chemical industries to decarbonize is exacerbated by the climate action agendas. From the perspective of industrial biotechnology, microorganisms that can grow using CO_2_ are of great interest due to their ability to channel cheap and abundant carbon feedstock toward more valuable compounds.^3^ This process allows conversion of CO_2_ into useful products while simultaneously reducing consumption of fossil fuels in the manufacturing process, thereby decreasing atmospheric carbon concentration. To fully realize such a system, several criteria must be fulfilled: (i) the organism(s) selected is capable of rapidly achieving high cell density in chemolithoautotrophic conditions, (ii) the organism(s) must be genetically amenable and robust in terms of genetic stability, and (iii) possess a simple metabolism which can be redirected toward other valuable compounds.^4^

*Cupriavidus necator* H16 is an industrially relevant and metabolically interesting organism due to its capacity to grow using CO_2_ and H_2_ as sole carbon and energy sources, respectively.^5^ It is well known for its ability to accumulate the biodegradable polymer polyhydroxybutyrate (PHB), a species of polyhydroxyalkanoate (PHA).^6^ Additionally, PHB levels over 80% have been achieved during chemolithoautotrophic growth, highlighting the ability of the organism to convert inorganic CO_2_ to organic carbon molecules.^7^ Production of PHB was reported in *C*. *necator* H16 is facilitated by the catalytic activities of the enzymes coded by the *phaCAB* operon, which begins with the condensation of 2 molecules of the central metabolite acetyl-CoA. Acetyl-CoA provides an ideal starting point to produce several commercially important compounds including 3-hydroxypropionate, fatty acids, diols, and polyketides.^8−13^ In addition to these chemicals, acetyl-CoA is also a key metabolite in the mevalonate biosynthetic pathway.^14^ As such, *C*. *necator* makes an ideal organism to produce mevalonate from CO_2_.

*C*. *necator* H16’s genetic stability or its robustness in maintaining expression vectors has been tested previously.^15^ Previous observations suggest that *C*. *necator* H16 is not capable of stably maintaining heterologous plasmids for many generations. Since similar results were obtained with plasmids possessing different replication origins, the inability to propagate expression vectors may be a general trait of *C*. *necator* H16. Genetic tools to improve plasmid stability in this species have already been reported in the literature and were based on antibiotic resistance,^16^ complementation of mutations in essential genes,^17^ and postsegregational killing.^18^ Although the plasmid stability was improved by these approaches, plasmid loss was still observed in *C*. *necator* H16. Since most of these systems were tested only under heterotrophic conditions, it is important to develop molecular tools capable of stabilizing plasmid-based expression systems under autotrophic conditions. To date, the only study focusing on the development of a plasmid addiction system that specifically works under autotrophic conditions was carried out by Lütte and co-workers.^19^ Such a tool was based on the *in trans* complementation of mutations in *hoxA*, the gene coding for the transcriptional factor responsible for activating the expression of hydrogenases in *C*. *necator* H16 and was successfully employed to improve heterologous production of cyanophycin, a branch polypeptide found mostly in cyanobacteria,^20^ from CO_2_ and H_2_. However, this plasmid addiction system presents some intrinsic limitations, such as the large size (∼9.1 Kb) of the construct used to complement Δ*hoxA*, which was shown to cause metabolic burden, thus affecting *C*. *necator* H16 growth,^19^ while also limiting the size of DNA cargos that can be cloned in the *hoxA*-complementation vector. In addition, this system is not capable of stabilizing plasmids under formatotrophic conditions, where H_2_ is replaced with formate as the electron donor. Since formate has recently been gaining popularity as a feedstock for microbial fermentations in the biotech industry sector,^21,22^ the demand for plasmid maintenance tools that are effective under these conditions is likely to increase in the future.

**Figure 1:**
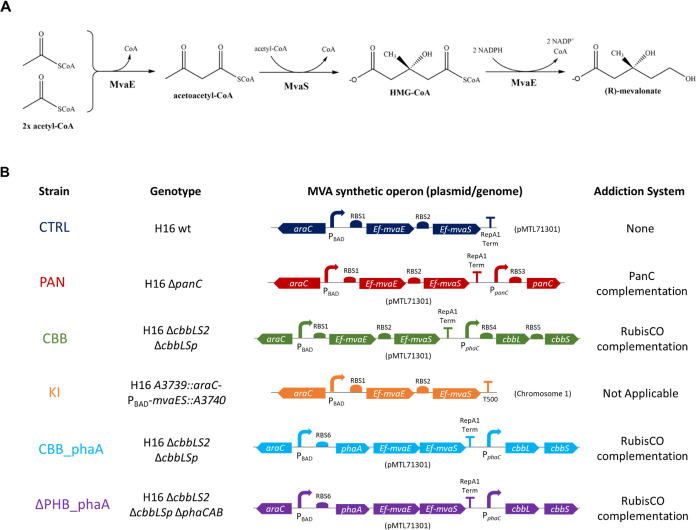
Schematic representation of the upper MVA pathway and summary of the *C*. *necator* H16 derivative strains used in this study. (A) The upper mevalonate pathway facilitates the conversion of three acetyl-CoA molecules to one molecule of (R)-mevalonate via a three-step pathway. The conversion is catalyzed by an acetyl-CoA acetyltransferase/HMG-CoA reductase (*Ef*-MvaE) and an HMG-CoA synthase (*Ef*-MvaS), where HMG stands for 3-hydroxy-3-methylglutarate. Both the *mvaE* and *mvaS* genes used in this study were obtained from *Enterococcus faecalis* (Ef) and their DNA sequences were codon-optimized for *Escherichia coli* (see the Supporting Information for more details). (B) *C*. *necator* H16 derivative names and genotypes, alongside a schematic representation of their corresponding MVA synthetic operon organization and localization (on plasmid/genome integrated) and the description of their addiction systems (where applicable), are reported. RBS1 and RBS2 are synthetic ribosome binding sites generated using the RBS calculator tool (Salis et al., 2009); RBS3 is the native RBS found upstream of the *C*. *necator* H16 *panC* gene; RBS4 is the *Cn*-*phaC* gene native RBS; RBS5 is the native RBS found upstream of the *Cn-cbbS2* gene in the *cbbLS2* operon located on *C*. *necator* H16 chromosome 2; RBS6 is the *Cn*-*phaA* gene native RBS; Term is the Rho-independent terminator found downstream of the *Clostridium pasteurianum fdx* gene; T500 is a synthetic terminator from Yarnell and Roberts.^48^

In this study, we aimed to produce a commercially interesting compound from CO_2_ using *C*. *necator* H16 strains equipped with stable multicopy extra-chromosomal expression systems as microbial factories. To this end, two metabolic plasmid addiction systems were developed to overcome plasmid instability issues, which are known to affect productivity in fermentative reactions, even in the presence of antibiotics.^23,24^ Stable multicopy plasmids display important advantages over chromosomal integrations, which are difficult to construct and are associated with decreased protein expression due to low copy numbers of the genes of interest, leading to reduced product synthesis.^17^ Hence, we investigated essential-gene complementation as a potential method to create stable episomal expression systems for *C*. *necator* H16. *panC* (Pantothenate synthetase)^25^ and *cbbLS* (Ribulose bisphosphate carboxylase large and small subunits)^26^ based complementation systems were developed and tested for their ability to improve plasmid stability in *C*. *necator* H16, under autotrophic conditions. Both the *panC* gene and the cbbLS are essential, with the latter under autotrophic conditions. To test these systems, we chose the mevalonate (MVA) biosynthetic pathway (Figures 1A and S1 in the Supporting Information) as an example, due to the absence of literature reports demonstrating production of this molecule in *C*. *necator* H16, in gas fermentation. The mevalonate pathway is used by eukaryotes, archaea, and certain bacteria to convert acetyl-CoA to mevalonate,^27^ a precursor for isoprenoids, a class of molecules that include cholesterols, steroids, and cell wall components.^28^ Moreover, mevalonate has commercial applications in the medical, materials, cosmetics, and food industry sectors.^27−33^ MVA biosynthesis from organic carbon sources such as glucose, glycerol, and acetate have already been described.^14,34−41^ While *C*. *necator* H16 can grow on organic carbon sources (preferably fructose and gluconate), these are not cost-effective for large-scale production of cheap feedstock materials.^42^ Aside from the environmental benefit of carbon sequestration, mevalonate was produced from CO_2_ using *C*. *necator* H16 would result in a more sustainable and economically viable process. Here, we report the development of a robust and reliable plasmid addiction tool to stabilize extra-chromosomal expression systems under autotrophic conditions. Using this tool, we successfully produced significant amounts of MVA (6 atoms of carbon; C6) from the C1 GHG CO_2_, employing engineered *C*. *necator* H16 strains as microbial cell factories. The results presented here contribute to expand the list of platform/value-added chemicals, which already included solvents, fuels, acids, and biopolymer precursors, produced from CO_2_ using *C*. *necator* H16.^8,11,43−48^

## 2 Materials and Methods

### 2.1 Bacterial Strains, Plasmids, and Culture Conditions

*E*. *coli* DH5α (Invitrogen, Carlsbad, CA) was used for general cloning and plasmid propagation. *E*. *coli* S17–1 (Invitrogen, Carlsbad, CA) was used for the conjugative transfer of plasmids. *E*. *coli* strains were grown at 37 °C in Lysogenyl Broth (LB) medium. For *E*. *coli* strains carrying a plasmid, the medium was supplemented with 15 μg/mL tetracycline. *C*. *necator* H16 strains were grown at 30 °C in LB or mineral salts medium (MM)^49^ supplemented with 10 μg/mL gentamycin. Sodium gluconate was used as the carbon source at a concentration of 0.4% (w/v).^50^ Cultivation of *C*. *necator* H16 strains auxotrophic for pantothenate was achieved by supplementing the culture media with 1 mM pantothenic acid. The full lists of bacterial strains, plasmids, and primers used in this study are reported in Tables S1 and S2 (Supporting Information). Schematic representations of the upper part of the MVA biosynthetic pathway and of the *C*. *necator* H16 derivatives are shown in Figure 1.

### 2.2 Culture Media Composition

The Nitrogen-limited MM used during the flask cultivation experiments carried out to produce MVA under heterotrophic conditions was prepared as described by Schlegel et al.^49^ except for NH_4_Cl, which was not provided. This was supplemented with 2.5% (w/v) fructose, and pH was adjusted to 6.9. The 80 mM Na-formate MM used to cultivate *C*. *necator* H16 derivatives under organo-autotrophic conditions was prepared as described previously.^51^ Na-formate was used to provide both the energy and carbon sources for the host and for the induction of the CBB pathway for subsequent autotrophic cultivation with CO_2_ and H_2_. The culture medium used in the autotrophic fermentation experiments was a modified version of DSMZ81, as previously described.^8^

### 2.3 Construction of Plasmids Used in This Study

Gene deletions and expressions were achieved using plasmids belonging to the SBRCpMTL7000 modular vector series, as described by Ehsaan et al.^15^ Deletion of target genes is in *C*. *necator* H16 was facilitated via pMTL70621-SacB, a suicide modular vector specifically developed for *C*. *necator* H16, also described by Ehsaan et al.^15^ This vector harbors the *tetA* tetracycline-resistance marker from plasmid pBR322 and the *E*. *coli*-specific ColE1 replicon (nonfunctional in *C*. *necator* H16) and the *oriT* origin of transfer, also from pBR322, and the *sacB* counter-selection marker from plasmid pLO3.^52^ Expression of the mevalonate production pathway was carried out using the pMTL71301 modular shuttle vector,^15^ which carries the *oriV* origin of replication, the mobilization (*mob*) gene and the *tetA/R* tetracycline-resistance marker from plasmid pBBR1MCS3. The average copy number per cell of this plasmid is 42.57 ± 2.00 in *C*. *necator* H16, as previously determined.^15^ The deletion and integration plasmids used in this study (pMTL70621::Δ*panC*, pMTL70621::Δ*cbbLS*2 and pMTL70621::Δ*cbbLS*p; pMTL70621::KI_A3739::*araC-*P_BAD_-*mvaES*::A3740) were derived from pMTL70621-SacB. The plasmids for complementation of the Δ*panC* and *Δ*Δ*cbbLS* mutations (pMTL71301::*panC* and pMTL71301::T500-P_*phaC*_-*cbbLS*2), as well as the MVA pathway expression plasmids (pMTL71301::*araC*-P_BAD_-*mvaES*, pMTL71301::*araC*-P_BAD_-*mvaES*::*panC*, pMTL71301::*araC*-P_BAD_-*mvaES*-P_*phaC*_-*cbbLS* and pMTL71301::*araC*-P_BAD_-*phaAmvaES*-P_*phaC*_-*cbbLS*) were derived from pMTL71301. All plasmids were delivered to *C*. *necator* H16 by conjugal transfer, using *E*. *coli* S17–1 as donor strain, as previously described.^52^ Transconjugants were selected on solid MM supplemented with 0.4% sodium gluconate and 15 μg/mL tetracycline. The cloning strategies used for the construction of the plasmids used in this study are described in detail in the Supporting Information.

All primers were ordered from Sigma-Aldrich. The DNA parts required for plasmid construction were amplified using Q5 High-Fidelity polymerase (New England Biolabs), while diagnostic polymerase chain reaction (PCR) reactions were carried out using OneTaq Quick Load polymerase (New England Biolabs). Molecular cloning was carried out using either conventional restriction-ligation or NEBuilder HiFi DNA assembly (New England Biolabs) techniques. Following cloning, the DNA constructs of interest were verified using the Eurofins Genomics Sanger sequencing service.

### 2.4 Construction of the H16 Δ*panC* and H16 *Δ*Δ*cbbLS* Deletion Mutants

Plasmids pMTL70621::Δ*panC* and pMTL70621::Δ*cbbLS*2 were transformed into *E*. *coli* S17–1 to allow their conjugative transfer to *C*. *necator* H16, which was carried out as mentioned in paragraph 2.3. Allelic exchange was obtained using a *sacB* counter-selection method, as previously described.^52^ Screening for the clones that underwent the second crossover event was achieved by replica plating of selected colonies on plain LB and LB + 15 μg/mL tetracycline plates. The tetracycline-sensitive colonies were then screened by PCR using primer pairs annealing just outside of the homology arms used to construct the in-frame gene deletions (panC_EXT_FW + panC_EXT_REV for Δ*panC* and cbbLSch_OUT_FW + cbbLSch_OUT_RV for Δ*cbbLS*2). The colonies giving amplicons of the expected size were then selected, and deletion of the genes of interest was confirmed using primer pairs annealing within the deleted DNA sequences (panC_INT_FW + panC_INT_REV) for Δ*panC* and cbbLSch_IN_FW + cbbLSch_IN_RV for Δ*cbbLS*2. In the case of the Δ*panC* mutant, due to the expected auxotrophy of this strain, screening was carried out using LB plates supplemented with 1 mM pantothenate. To confirm the pantothenate auxotrophy of the *C*. *necator* H16 Δ*panC* mutant; this was streaked on LB and MM plates, in the presence and absence of pantothenate, and incubated at 30 °C for 2 weeks. To construct the *C*. *necator* H16 *Δ*Δ*cbbLS* mutant the process described above was repeated by conjugating the pMTL70621::Δ*cbbLS*p plasmid into the *C*. *necator* H16 Δ*cbbLS*2 strain. Inactivation of the *cbbLS*p gene was verified by PCR, using primer pairs cbbLSm_OUT_FW + cbbLSm_OUT_RV and cbbLSm_IN_FW + cbbLSm_IN_RV.

### 2.5 Integration of the *araC*-P_BAD_-*mvaES_*T500 Operon in *C*. *necator H16*

To construct the *C*. *necator* H16 strain carrying the genomic integration of the MVA synthetic pathway (KI), plasmid pMTL70621::KI_A3739::*araC-*P_BAD_-*mvaES*::A3740 was transformed in *E*. *coli* S17–1 and conjugated into *C*. *necator* H16, as described in paragraph 2.3. To screen for the successful integration of the *araC-*P_BAD_-*mvaES_*T500 operon in the intergenic region between genes H16_A3739 and H16_A3740, primer pairs composed of 1 primer annealing outside of the homology arms and 1 primer annealing within the *araC-*P_BAD_-*mvaES_*T500 DNA sequence were used. These were the following: A3739_EXT_FOR + JW5seq3_REV2 and JW5seq6_FOR + A3740_EXT_REV.

### 2.6 Autotrophic Cultivation Setup in Bioreactors

Precultures of strains CTRL, PAN, CBB, KI, and ΔPHB_phaA were set up in 500 mL baffled flasks (3 flasks for each strain), containing 100 mL of YLB + 10 μg/mL gentamycin and 15 μg/mL tetracycline. The cultures were then incubated at 30 °C for 24 h with shaking (200 rpm). Seed cultures of CBB_phaA, instead, were prepared by inoculating this strain in 250 mL baffled flasks (10 flasks per strain), containing 50 mL of 80 mM Na-formate MM and 10 μg/mL gentamycin and 15 μg/mL tetracycline. The flasks were then incubated at 30 °C for 48 h, with shaking (200 rpm), in an atmosphere composed of 10% CO_2_ and 90% air. In all cases, the total biomass produced was harvested by centrifuging the bacterial cultures in 50 mL Falcon tubes at 8000 rpm for 5 min. Cell pellets were then washed twice in 25 mL of gas fermentation medium and finally resuspended in a total of 100 mL of the same culture medium before being inoculated in the bioreactors.

Batch cultivations under autotrophic conditions were performed in a DASGIP Bioreactor System (Eppendorf), using 1 L capacity vessels with a working volume of 750 mL, as described by Bommareddy et al.^8^

### 2.7 Plasmid Stability Assay

Plasmid stability was assessed by withdrawing culture samples from the bioreactors at the time of inoculation, at the time of induction with 0.2% l-arabinose and every 24 h following this time, until the 168 h postinduction time point. These samples were then serially diluted with phosphate-buffered saline (PBS) and triplicates of each dilution were spot-plated (20 μL drops) on LB plates supplemented with 10 μg/mL gentamycin in the absence (LB) or presence of 15 μg/mL tetracycline (Tet). CTRL and PAN samples were also plated on LB+ gentamycin plates supplemented with 1 mM Pantothenate (Pan). Following 72 h of incubation at 30 °C, colonies were counted to determine the number of viable cells (cfu/mL) on each of the three different plate types for each strain at each time point. The viable counts data were used to assess plasmid stability by dividing the number of cfu/mL recorded on plates supplemented with tetracycline (Tet) by the viable counts on either LB or Pan.

### 2.8 Quantification of Gas Consumption, MVA and PHB Production

Consumption of CO_2_, O_2_, and H_2_ gases by the *C*. *necator* H16 derivatives used in this study was calculated based on the off-gas mixture composition, which was analyzed in real-time by an online multiplexed Raman Laser Analyzer (Atmospheric Recovery Inc.), as previously described.^8^ Culture samples collected from flasks and bioreactors were analyzed for the presence of MVA. Following centrifugation of samples at 14,000 rpm for 1 min, supernatant was collected, filter-sterilized, and transferred to clean tubes. An equivalent volume of the mobile phase, spiked with 50 mM valerate as an internal standard, was added to the supernatants. The mobile phase was composed of 0.005 M H_2_SO_4_. The mix was passed through a membrane filter with a pore size of 0.22 μm and transferred to HPLC autosampler glass vials. Samples were then analyzed using a Dionex 3000 HPLC system (Thermo Fisher Scientific) equipped with a Rezex ROA-organic acid H+ (8%) 150 mm × 7.8 mm × 8 μm column (Phenomex) and a diode array detector ultraviolet–visible (UV–vis 210 nm). The column was operated at 35 °C with a flow rate of 0.5 mL/min. Samples were run for 30 min, and the injection volume was 20 μL.

To determine biomass and PHB yields, samples of bacterial cultures (2 mL) collected from bioreactors at each time point were centrifuged for 1 min at 14,000 rpm and supernatants were analyzed by HPLC for quantification of MVA production, as described above and previously.^37−40^ Cell pellets were washed once with 1 mL of PBS before being lyophilized. The freeze-dried pellets were used to quantify the biomass concentration (CDW, g/L). Following CDW determination, cell pellets were analyzed by GC/MS (Agilent Technologies, GC 6890N, MS5 973N) to obtain PHB yields (CDW%), as previously described.^53^

## 3 Results and Discussion

### 3.1 Construction of a Plasmid Addiction System Based on the *In Trans* Complementation of a Pantothenate Auxotrophy

Since plasmid instability is one of the major factors hindering the application of *C*. *necator* H16 as microbial chassis for the expression of heterologous metabolic pathways in fermentation processes at industrial scale,^16,17,54,55^ we sought to develop a plasmid addiction system capable of improving plasmid stability in *C*. *necator* H16 under autotrophic conditions. One of the possible ways to create a plasmid addiction system is to complement an auxotrophic mutant by providing the missing biosynthetic gene on a plasmid.^23^ In this study, we identified the *panC* gene, encoding a pantothenate synthetase, as a potential addiction system candidate. Indeed, PanC catalyzes the conversion of β-alanine and pantoate into pantothenic acid (vitamin B5),^25^ as depicted in Figure S2. Pantothenate is an essential metabolite since it is an intermediate in Coenzyme-A biosynthesis and *panC* gene essentiality in *C*. *necator* H16 was also confirmed by a recently published TraDIS analysis.^56^ Consistently with these results is a *C*. *necator* H16 mutant carrying the in-frame deletion of the *panC* gene could only be obtained by supplementing the culture media with 1 mM pantothenate throughout the genetic modification procedure. Indeed, the *C*. *necator* Δ*panC* mutant is auxotrophic for pantothenate, as shown in Figure S3. We were able to demonstrate the ability of the *C*. *necator* H16 Δ*panC* mutant that could grow in the absence of pantothenate could be restored by transforming this strain with the pMTL71301::*panC* plasmid, carrying a functional copy of the *panC* gene under the control of its native promoter (Figure S3). To assess how the *panC*-based plasmid addiction system would perform in fermentation processes for the biosynthesis of heterologous metabolites of interest, the *araC*/P_BAD_-*mvaES* synthetic operon for the biosynthesis of MVA was cloned into pMTL71301::*panC*, thus obtaining pMTL71301::*araC*-P_BAD_-*mvaES*::*panC*. This plasmid was then transferred to the *C*. *necator* H16 Δ*panC* mutant by conjugation.

### 3.2 Test of the *panC*-Based Plasmid Addiction System in Autotrophic Batch Cultivation

**Figure 2:**
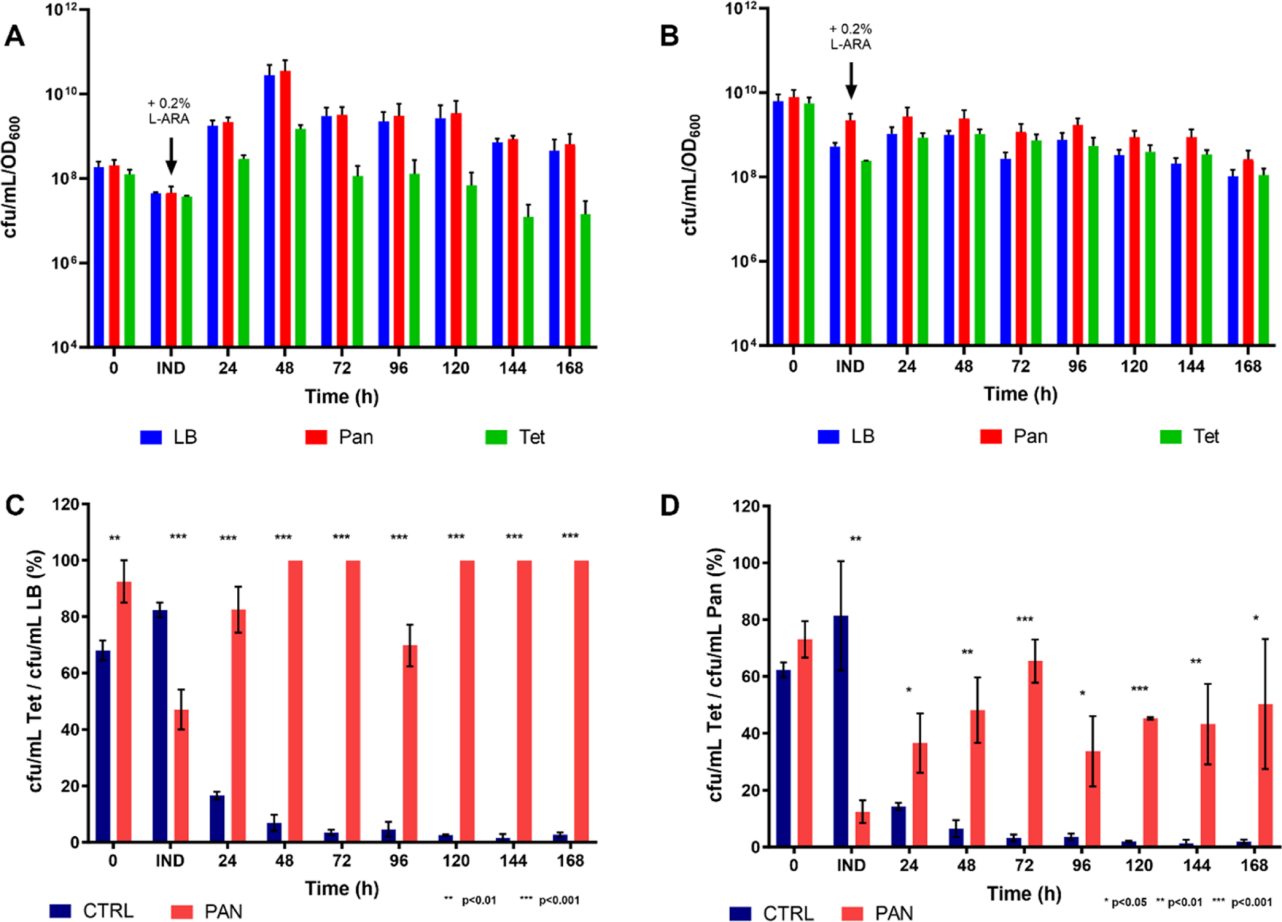
Viable counts and plasmid stability in CTRL and PAN. Number of cfu/mL normalized by the OD_600_ values (cfu/mL/OD_600_) generated by (A) *C*. *necator* H16/pMTL71301::*araC*-PBAD-*mvaES* (CTRL) and (B) *C*. *necator* H16 Δ*panC*/pMTL71301::*araC*-PBAD-*mvaES::panC* (PAN) on LB; Pan and Tet at the time of inoculation (*t* = 0 h), at the time of induction with 0.2% l-arabinose (IND) and at the 24, 48, 72, 96, 120, and 168 h postinduction time points and percentage of CTRL (dark blue) and PAN (red) cells retaining the plasmid calculated at the time of inoculation (0 h), at the time of induction with 0.2% l-arabinose (IND) and at the 24, 48, 72, 96, 120, and 168 h postinduction time points, as the ratios of (C) cfu/mL on Tet/cfu/mL on LB and (D) cfu/mL on Tet/cfu/ml on Pan. The mean values were calculated based on three biological replicates and the error bars represent standard deviation. Data were analyzed using the multiple *t* tests statistical method. *, **, and *** indicate statistically significant differences between the CTRL and PAN strains.

**Figure 3:**
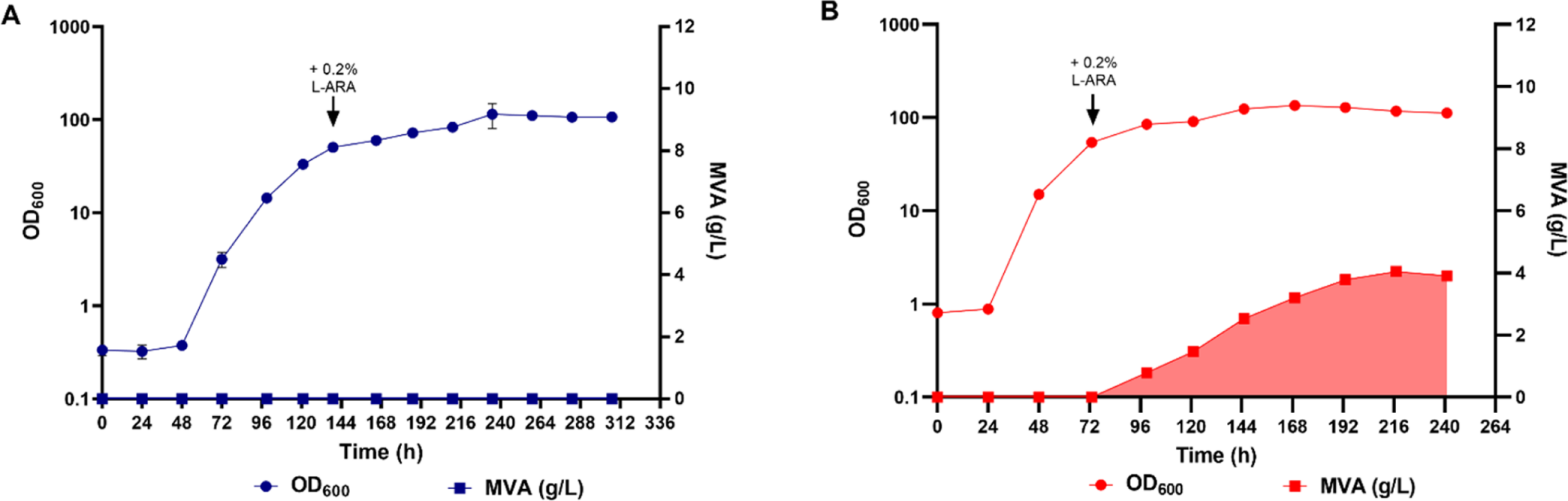
Growth and MVA production in CTRL and PAN. Round dots represent the average of 3 OD_600_ values measured at each time point, while squares correspond to the MVA titers (g/L) produced at each time point by (A) CTRL (dark blue) and (B) PAN (red). MVA production is highlighted by the shaded area. The times of induction with 0.2% l-arabinose are indicated by black arrows.

The *C*. *necator* H16 Δ*panC*/pMTL71301::*araC*-P_BAD_-*mvaES*::*panC* strain (from now on abbreviated as PAN), which harbors the modular vector carrying the synthetic pathway for MVA production and is equipped with the *panC*-based plasmid addiction system, was grown in the DASGIP parallel bioreactor system (Eppendorf) under chemolithoautotrophic conditions, using a gas mixture composed of 78% H_2_, 19% air and 3% (v/v) CO_2_. The control strain was *C*. *necator* H16/pMTL71301::*araC*-P_BAD_-*mvaES* (CTRL), which does not carry any plasmid addiction system, and was grown under the same experimental conditions. Culture samples were collected from the bioreactors at the time of inoculation (Day 0), at the time of induction with 0.2% l-arabinose and every 24 h following this time, until the end of the experiment. These were serially diluted in PBS and triplicates of each dilution were spot-plated (20 μL drops) on LB plates, either plain (LB) or supplemented with 1 mM Pantothenate (Pan) or 15 μg/mL tetracycline (Tet). Gentamycin (Gm; 10 μg/mL) was added to all plate types to reduce the risk of contamination by other bacterial species (e.g. *E*. *coli*) since *C*. *necator* H16 is naturally resistant to low concentrations of this antibiotic. Following 72 h of incubation at 30 °C, colonies were counted to determine the number of viable cells (cfu/mL) on each of the three different plate types, for each strain, at each time point. The numbers of cfu/mL were then normalized by the OD_600_ values recorded at each time point (cfu/mL/OD_600_) (Figure 3A,B). The viable counts data were used to assess plasmid stability by dividing the number of cfu/mL recorded on plates supplemented with tetracycline by the viable counts on either LB or Pan (Figure 2C,D).

As shown in Figure 2C, the *panC*-based plasmid addiction system appeared to increase plasmid stability since the percentage of tetracycline-resistant cells over the total number of cfu/mL yielded on LB by PAN is significantly higher than that of the control strain at every time point, except for the time of induction, at which the plasmid was significantly more stable in the CTRL strain. However, this analysis does not consider the presence of plasmid-free social cheaters in the population.^57^ Indeed, the pantothenate produced by “cooperators” (the cells still harboring the plasmid) diffuses in the culture media, where it becomes a “public good” that can be exploited by “social cheaters”, which can therefore survive without the plasmid expressing the *panC* gene. These cheaters are still auxotrophic for pantothenate and cannot grow in the absence of this metabolite. Hence, culture samples were also plated in the presence of pantothenate to obtain the total number of viable PAN cells at each time point and calculate the actual portion of PAN cells still retaining the plasmid. Figure 2D shows that when the presence of social cheaters is considered, the *in trans* complementation of the Δ*panC* mutation was unable to improve plasmid stability reliably and consistently in *C*. *necator* H16 since the portion of PAN cells retaining the plasmid varied dramatically throughout the fermentation time course, getting as low as ∼12% at the time of induction.

### 3.3 MVA Production from CO_2_ in PAN and CTRL

Since the primary aim of this study is to develop plasmid addiction systems that could be employed in fermentation processes for the biosynthesis of chemicals of interest from CO_2_ using *C*. *necator* H16 as the microbial chassis, the ability of the CTRL and PAN strains to produce MVA under autotrophic conditions was tested. Growth of these strains was monitored by measuring OD_600_ offline using a standard spectrophotometer. When the OD_600_ of the cultures reached a value of at least 50 (51 for CTRL and 54 for PAN), the expression of the *mvaES* operon was induced by supplementing 0.2% l-arabinose in the culture media. Culture samples were collected at the time of induction and every 24 h for the following 168 h. These were centrifuged and the culture supernatants were filtered and analyzed by HPLC. Culture pellets were frozen and used for CDW and PHB quantification. The growth curves and MVA titers produced over time by the CTRL and PAN strains are shown in Figure 3.

It is interesting to note that the CTRL strain displayed a significantly longer lag phase than did PAN (48 and 24 h, respectively). The control strain also grew slower than the one carrying the *panC*-based plasmid addiction system, suggesting that the presence of the *panC* gene on a multicopy plasmid may result in increased production of pantothenate and Coenzyme-A, thus positively affecting biomass formation. Importantly, the control strain did not produce any MVA. This was surprising, since plasmid stability data showed that about 80% of the total CTRL population had retained the plasmid until the time of induction. However, this rapidly decreased following l-arabinose supplementation and became negligible by the 72 h postinduction time point (Figure 2C,D). On the other hand, production of up to 4 g/L MVA was observed in the PAN strain (Figure 3B), suggesting that the *panC*-based plasmid addiction system was able to improve MVA production in *C*. *necator* H16. Nevertheless, our data provide evidence in support of a correlation between pantothenate overproduction and the emergence of subpopulations of plasmid-free PAN cells, which may significantly reduce the overall efficiency of fermentation processes (see the Supporting Information for more details). Therefore, the *panC*-based plasmid addiction system was deemed unsuitable for implementation in large-scale fermentation processes, employing *C*. *necator* H16 as a microbial chassis.

### 3.4 Construction of a Plasmid Addiction System Based on the *In Trans* Complementation of *C*. *necator* H16 RubisCO Null Mutants

To avoid the shortfall associated with the *panC*-dependent tool, we focused on developing a plasmid addiction system based on the complementation of a “private good”, which is not secreted in the culture media. RubisCO, which is central to the CO_2_ fixation in *C*. *necator* H16, was identified as a good candidate for this purpose. Being one of the key enzymes of the CBB cycle, RubisCO is likely to be essential for the growth of *C*. *necator* H16 under autotrophic conditions. In support of this hypothesis, data previously obtained in our laboratory indicate that *C*. *necator* H16 mutants lacking both chromosomal and megaplasmid-borne copies of the RubisCO-encoding genes (*cbbLS*) are unable to grow using CO_2_ as the sole carbon source. Moreover, our data suggest that the ability of *C*. *necator* H16 RubisCO null mutants to grow under autotrophic conditions can be restored by introducing the *cbbLS* genes on a multicopy plasmid, under the control of the medium-strength constitutive promoter P_*phaC*_ ().^58^ Therefore, the P_*phaC*_*-cbbLS* synthetic operon was cloned in the pMTL71301::*araC*-PBAD-*mvaES* vector. The resulting plasmid was transferred to the *C*. *necator* H16 *Δ*Δ*cbbLS* mutant, lacking both copies of the *cbbLS* genes, to obtain the H16 *Δ*Δ*cbbLS*/pMTL71301::*araC*-PBAD-*mvaES*::P_*phaC*_*-cbbLS* (CBB) strain.

### 3.5 Test of the *cbbLS*-Based Plasmid Addiction System in Autotrophic Batch Fermentation

**Figure 4:**
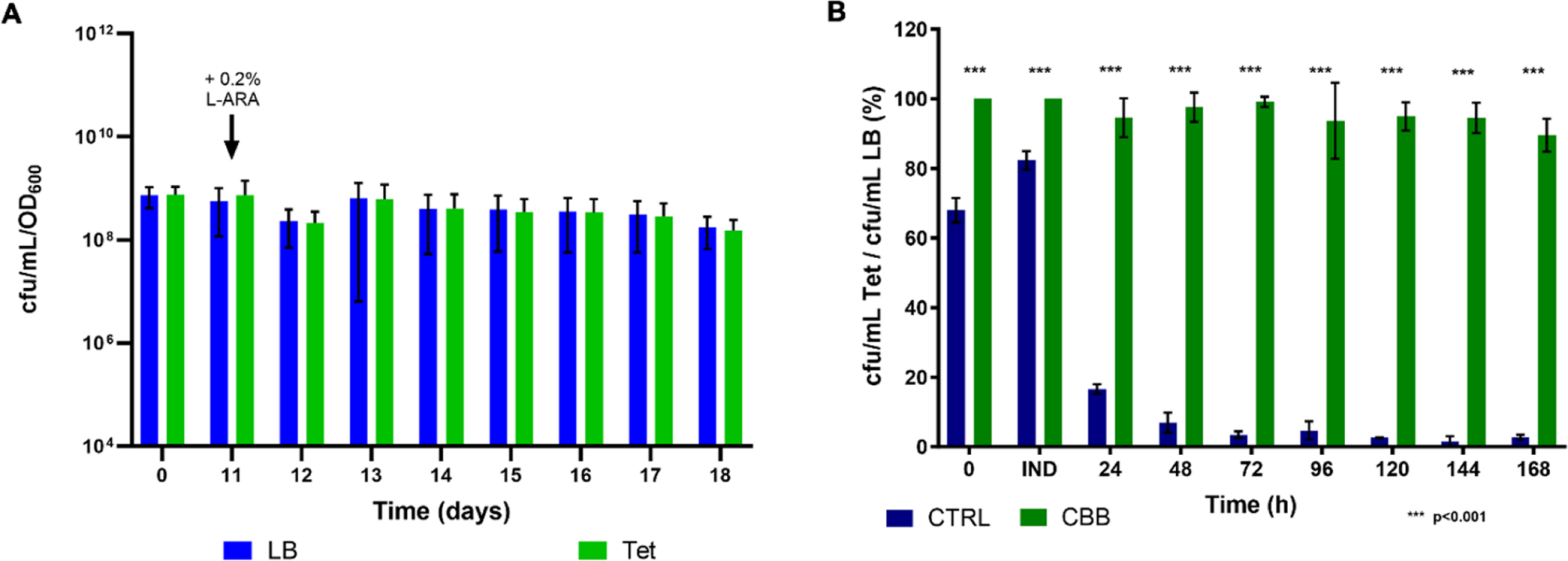
Viable counts and plasmid stability in CBB strain. (A) Number of cfu/mL normalized by the OD_600_ values (cfu/mL/OD_600_) generated by the CBB strain on LB and Tet at the time of inoculation (*t* = 0 h), at the time of induction with 0.2% l-arabinose (IND) and at the 24, 48, 72, 96, 120, and 168 h postinduction time points and (B) percentage of CTRL (dark blue) and CBB (dark green) cells retaining the plasmid calculated at the time of inoculation (0 h), the time of induction (IND) and at 24, 48, 72, 96, 120, and 168 h after induction as the ratios of cfu/mL Tet/cfu/mL on LB. The mean values were calculated based on three biological replicates, and the error bars represent standard deviation. The data were analyzed using the multiple *t* tests statistical method. *** indicates statistically significant differences between the CTRL and CBB strains.

The CBB strain was cultivated in the DASGIP fermentation system using the same experimental conditions described in Section 3.2. Culture samples were collected from the fermenters at the time of inoculation (Day 0), at the time of induction with 0.2% l-arabinose and every 24 h for the 168 h following this time. The cfu/mL/OD_600_ data were obtained as described in Section 3.2. The results obtained are shown in Figure 4A. Plasmid stability in the CBB strain was calculated by dividing the amount of cfu/mL obtained on plates supplemented with tetracycline by the viable counts on the LB. In Figure 4B, these data are compared to those obtained with the CTRL strain.

As shown in Figure 4B, the *in trans* complementation of the RubisCO-encoding genes *cbbLS* dramatically and consistently improved the plasmid stability in *C*. *necator* H16. The percentage of tetracycline-resistant CBB cells remained over 90% of the total population throughout the duration of the batch fermentation experiment, which lasted 18 days. These values were significantly higher than those observed with CTRL strain at every time point. To the best of our knowledge, this is by far the most stable plasmid addiction system to date demonstrated in *C*. *necator* H16, particularly under autotrophic growth conditions.

### 3.6 MVA Production from CO_2_ by CBB and Comparison with a Strain Carrying the Genomic Integration of MVA-Producing Genes

**Figure 5:**
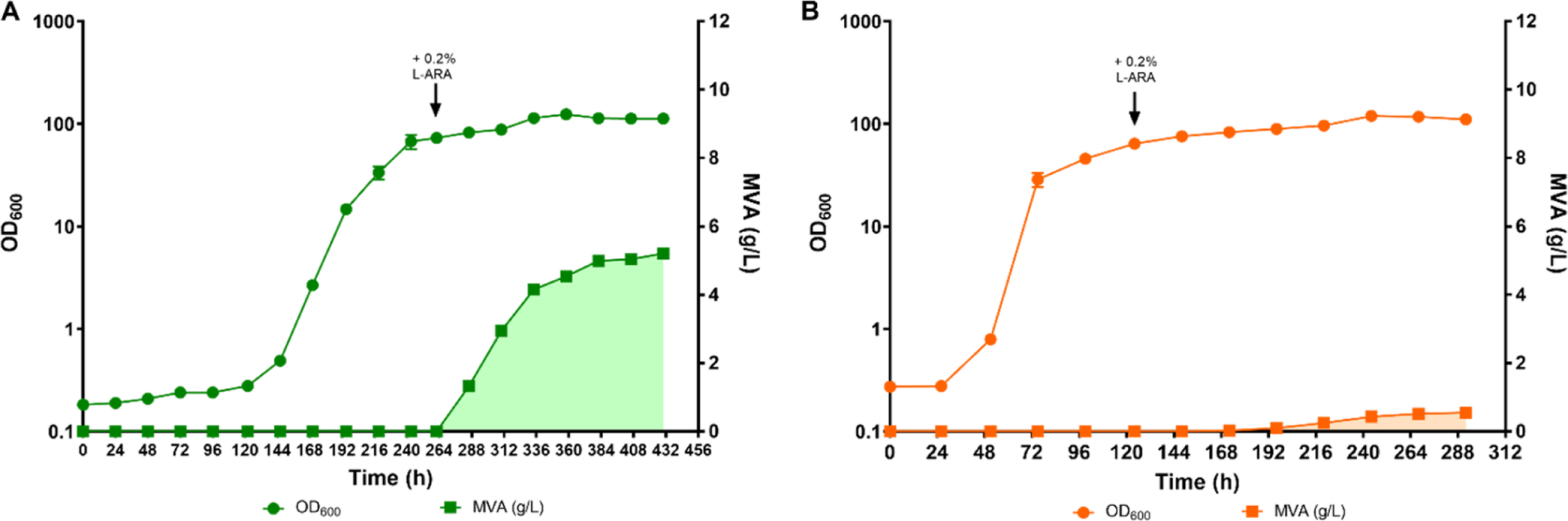
Growth and MVA production by the CBB and KI strains. Round dots represent the average of 3 OD_600_ values measured at each time point, while squares correspond to the MVA titers produced at each time point by (A) CBB (dark green) and (B) KI (orange). MVA production is highlighted by the shaded area. Times of induction with 0.2% l-arabinose are indicated by black arrows.

Chromosomal integrations of heterologous pathways have been shown to improve the genetic stability and have thus been regarded as a possible solution for plasmid instability. However, these may be difficult to construct and are often associated with reduced product yields, with respect to multicopy plasmid systems. Hence, a strain carrying the *araC*/P_BAD_-*mvaES* synthetic operon integrated in the genome was constructed. A neutral site for integration was selected among the *C*. *necator* H16 genomic regions that were classified as nonessential in the previously cited TraDIS analysis.^54^ The most suitable site was identified at the level of the intergenic region located between genes H16_A3739 and H16_A3740, encoding an AraC family transcriptional regulator and a 4-hydroxyacetophenone monooxygenase, respectively. This location was chosen also because of its proximity (∼7 kb) to the chromosome 1 origin of replication, which has been shown to increase gene copy numbers per cell (≥2) during exponential growth phase.^59^ The resulting strain, *C*. *necator* H16 *H16_A3739::araC*-P_BAD_-*mvaES::H16_A3740* (KI), and CBB were grown under autotrophic conditions to assess their MVA production performances, as described in Section 3.3. The results obtained are reported in Figure 5.

As shown in Figure 5A, the CBB strain was able to produce up to 5.2 g/L MVA, a significantly higher titer than that obtained with the PAN strain. Moreover, this MVA concentration was ∼10-fold higher than that produced by the KI strain (0.55 g/L), confirming that expression of heterologous pathways from stable multicopy plasmid systems results in considerably higher titers and yields of the product of interest. In addition, the RubisCO-based plasmid addiction system allowed for MVA to be produced continuously, from the time of induction to the end point of the fermentation, unlike what was observed with the strain carrying the *panC*-based addiction system. This observation, in combination with the plasmid stability data shown in Figure 4B, strongly indicates that the RubisCO-based addiction system meets the quality standards to be implemented in autotrophic fermentation processes involving microbial chassis carrying plasmid-borne metabolic pathways of interest, even at larger scales.

### 3.7 Effects of Co-Overexpressing PhaA and MvaES on MVA Production

**Figure 6:**
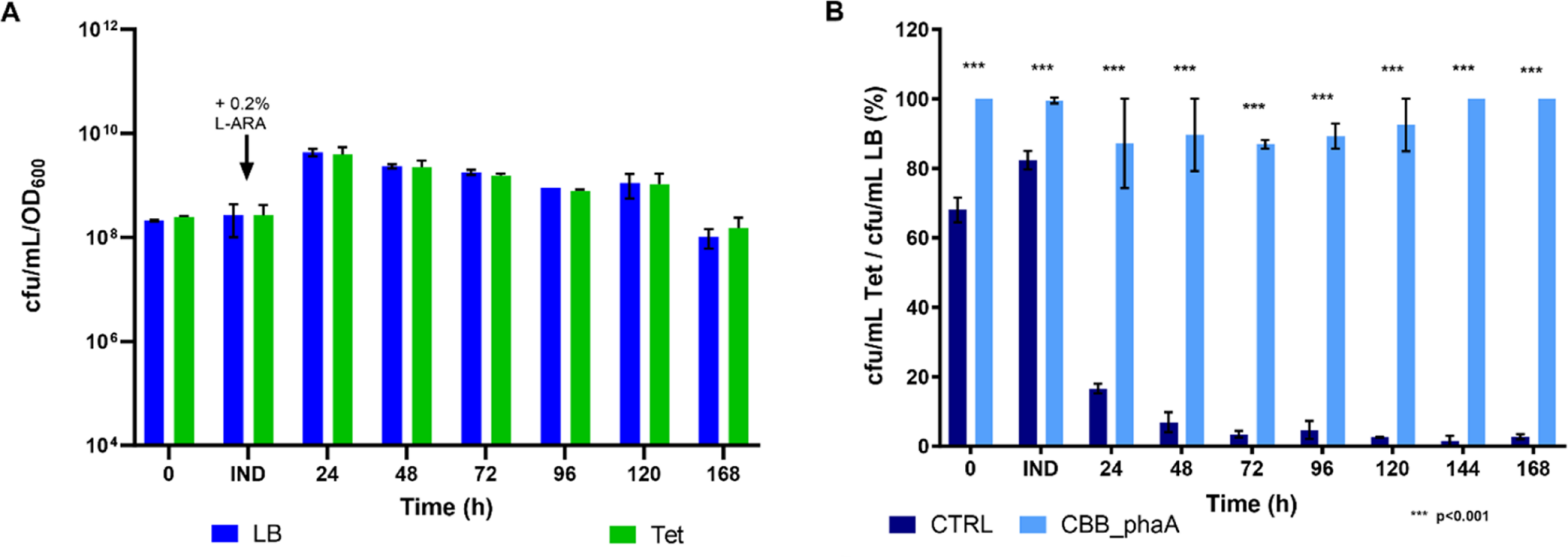
Viable counts and plasmid stability in the CBB_phaA strain. (A) Number of cfu/mL normalized by the OD_600_ values (cfu/mL/OD_600_) generated by the CBB_phaA strain on LB and Tet at the time of inoculation (*t* = 0 h), at the time of induction with 0.2% l-arabinose (IND) and at the 24, 48, 72, 96, 120, and 168 h postinduction time points and (B) percentage of CTRL (dark blue) and CBB_phaA (light blue) cells retaining the plasmid calculated at the time of inoculation (0 h), the time of induction (IND) and at 24, 48, 72, 96, 120, and 168 h after induction as the ratios of cfu/mL on Tet/cfu/mL on LB. The mean values were calculated based on three biological replicates and the error bars represent standard deviation The data were analyzed using the multiple *t* tests statistical method. *** indicates statistically significant differences between the CTRL and CBB_phaA strains.

**Figure 7:**
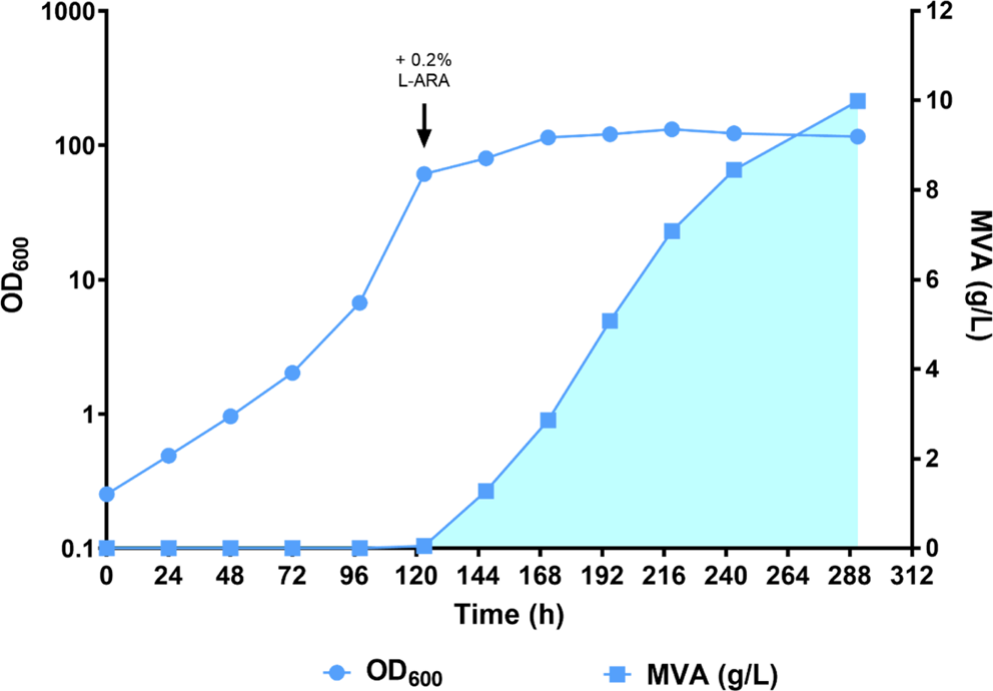
Growth and MVA production were calculated by CBB_phaA. Light blue dots represent the average of 3 OD_600_ values measured at each time point, while light blue squares correspond to the MVA titers produced at each time point by CBB_phaA. MVA production is highlighted by the shaded area. The time of induction with 0.2% l-arabinose is indicated by the black arrow.

In an attempt to further improve MVA production, the effects of overexpressing the *phaA* gene in the CBB strain genetic background were tested. *phaA* codes for an acetyl-CoA acetyltransferase (β-ketothiolase) that is native to *C*. *necator* H16, where it is involved in PHB biosynthesis alongside PhaB (acetoacetyl-CoA reductase) and PhaC (poly(3-hydroxyalkanoate polymerase)). The *Cn*-PhaA (native to *Cupriavidus necator*) enzyme has a significantly higher affinity for acetyl-CoA than *Ef*-MvaE (native to *E. faecalis)*(Km values of 390 μM and 600 μM, respectively).^60−62^ Hence, *Cn*-PhaA can catalyze the condensation of 2 acetyl-CoA molecules into acetoacetyl-CoA (the first reaction of the MVA pathway—Figure 1A) more efficiently than *Ef*-MvaE. Therefore, the *phaA* gene was cloned upstream of *mvaE* in pMTL71301::*araC*-Pbad-*mvaES*::*cbbLS* and the resulting plasmid (pMTL71301::*araC*-Pbad-*phaAmvaES*::*cbbLS*) was transformed in CBB to obtain strain CBB_phaA. Plasmid stability (Figure 6) and MVA production (Figure 7) by CBB_phaA were again tested in batch autotrophic fermentation, as described in Sections 3.2 and 3.3.

As can be seen in Figure 7, the CBB_phaA strain did not show any lag phase after being inoculated in the fermenter. This is because the seed cultures of this strain were grown under chemoautotrophic conditions (80 mM Na-formate MM, in the presence of 10% CO_2_) instead of the YLB complex medium used to preculture all of the other strains used in this study. Even though CBB_phaA reached an optical density (OD_600_ = 61) high enough to be induced with 0.2% l-arabinose sooner than the CTRL (∼5 vs ∼6 days—Figure 4A), the two strains showed comparable growth rates (0.045 Vs 0.052 h^–1^, respectively) when the lag phase displayed by CTRL was not considered. These data suggest that constitutive expression of the *cbbLS* genes from a multicopy plasmid does not appear to enhance the ability of *C*. *necator* H16 to grow under autotrophic conditions. Indeed, strain CBB also showed a similar growth rate (Figure 5A, μ = 0.043 h^–1^ between the 144 and 259 h time points). In terms of MVA production, CBB_phaA performed significantly better than the CTRL, PAN, and CBB strains, yielding a maximum MVA titer of approximately 10 g/L. The RubisCO-based plasmid addiction system significantly outperformed the *panC*-based one also in terms of MVA productivity. Indeed, the highest MVA productivity recorded with CBB_phaA was 0.092 g/L/h, which is over 2-fold higher than that observed with the PAN strain. This significant increase in MVA production efficiency must be largely attributed to the improved plasmid stability in CBB_phaA, with respect to PAN. However, introduction of the *phaA* gene in the *araC*-P_BAD_-*mvaES* synthetic operon also had a positive effect on MVA production, as indicated by the ∼2-fold increase in MVA titers observed in CBB_phaA, with respect to its isogenic strain CBB, in which PhaA was not overexpressed. As described for CBB, the CBB_phaA strain was also able to produce MVA until the end of the fermentation, even though productivity decreased in the last two 24 h time intervals to 0.044 and 0.020 g/L/h, respectively. Overall, these observations remark on the reliability of the RubisCO-based addiction system and further highlight its potential to be employed in larger-scale gas fermentation processes.

Since acetyl-CoA and acetoacetyl-CoA are intermediates of both the MVA and PHB biosynthetic pathways, the effects of knocking out PHB production on MVA synthesis were investigated. Interestingly, deleting the *phaCAB* operon in the CBB_phaA genetic background strongly and negatively affected MVA production in the resulting strain ΔPHB_phaA, which only synthesized up to 0.46 g/L MVA (Figure S5). This result was unexpected since the inactivation of PHB biosynthesis should have increased acetyl-CoA flux toward MVA production. However, it must be considered that the Δ*phaCAB* mutation may have impaired ΔPHB_phaA growth, as suggested by the peak OD_600_ value reached by this strain (∼16, Figure S5), which was significantly lower than those observed with PHB-producing strains (peak OD_600_ > 100). Although this difference in OD_600_ is in large part due to the reduced size of ΔPHB_phaA cells, resulting from the lack of intracellular PHB granules, it is highly likely that there is an absence of PHB production, which acts as the major carbon and redox sink for overflow metabolism in *C*. *necator* H16, significantly reduced the demand for CO_2_ and H_2_. This is supported by the observation that the amounts of CO_2_ consumed by ΔPHB_phaA after induction with l-arabinose were between 5.5- and 11.5-fold lower, with respect to the PHB-producing strains (Tables S3 and S4).

### 3.8 Comparison of Gas Uptake Rates, MVA, and PHB Production in the Different Strains

The amounts of CO_2_, O_2_, and H_2_ consumed by the CTRL, PAN, CBB, KI, CBB_phaA, and ΔPHB_phaA strains during each of the time intervals in between two consecutive culture sample collections are reported in Figure S6. It is very interesting to note that the CTRL strain consumed significantly lower amounts of each gas (CO_2_, O_2_, and H_2_), with respect to PAN and CBB_phaA. In terms of CO_2_ utilization, all of the *C*. *necator* H16 strains tested in these experiments, apart from ΔPHB_phaA, consumed similar amounts of CO_2_ up to the time of induction with 0.2% l-arabinose. This is not surprising since the strains were allowed to reach similar biomass levels (OD_600_ > 50) before inducing the expression of the *mvaES* genes, while the ΔPHB_phaA strain was induced at OD_600_ ∼ 10. However, the CO_2_ consumption rates of these strains differed significantly. PAN was the fastest strain to reach the target OD_600_ for induction in just 73 h (with respect to the 139 h required by the CTRL strain) and, consistently, showed the highest preinduction CO_2_ uptake rates (CUR) and growth rate (μ = 0.084 h^–1^ between *t* = 24 and *t* = 73 h) when the lag phase is not considered. Strains KI and CBB_phaA also took less time than CTRL to reach the target OD_600_ values (123 and 124 h, respectively). However, no significant changes in growth rates were observed. Differences in postinduction CUR were also observed among the strains, with the lowest rates displayed by CTRL and KI. This correlates well with the reduced MVA production in these strains. However, the significant differences observed in CUR are likely to be the result of a combination of factors, besides MVA biosynthesis. For instance, in the case of PAN, it could be speculated that the additional carbon uptake may have been employed to sustain an increased production of pantothenate, with respect to the control strain, due to the presence of multiple copies of the *panC* gene in each cell. Similarly, the CBB_phaA strain carries the *cbbLS* RubisCO-encoding genes on the same multicopy plasmid. Moreover, these genes are under the control of a medium-strength constitutive promoter instead of their native, highly regulated promoter. Hence, the consequent RubisCO overexpression may have artificially increased the demand for CO_2_, ultimately decreasing CO_2_ uptake rate via the CBB cycle. However, it must be pointed out that the levels of CO_2_ consumed following induction by the CBB strain, which differs from CBB_phaA only for not carrying a plasmid-borne copy of the *phaA* gene, were around 2-fold lower than in CBB_phaA and comparable to those displayed by the CTRL strain. Therefore, the significant increase in CO_2_ consumption observed in strain CBB_phaA appears to depend mostly on the overexpression of PhaA, resulting in high intracellular levels of acetoacetyl-CoA, which are then channeled toward both MVA and PHB biosynthesis.

Consumption of O_2_ and H_2_ were also significantly higher in PAN and CBB_phaA, with respect to the CTRL strain (Figure S6). The major differences were observed after induction, with postinduction H_2_ and O_2_ levels consumed by the *panC-* and RubisCO-complemented mutants being 30–35 and ∼56% higher than those of the control, respectively (Table S3). As already mentioned for CO_2_ utilization, the increased consumption levels for these gases may reflect the metabolic burden generated by overexpression of the *panC* and *phaA/cbbLS* genes in these strains. In addition, increased H_2_ consumption may have also been observed because biosynthesis of MVA (C_6_H_12_O_4_) and, in the case of PAN, pantothenic acid (C_9_H_17_NO_5_) both require reducing equivalents in the form of NADPH. Another possible explanation for the increased gas consumption in the strains carrying the *panC*- and RubisCO-based plasmid addiction systems, with the latter showing this behavior only when combined with *phaA* overexpression, may be found in the overproduction of PHB. Indeed, the biosynthesis of this polymer is a major NADPH-demanding process in *C*. *necator* H16. To investigate this possibility, biomass concentrations (CDW, g/L) and PHB yields (% of CDW) produced over time by the strains tested in this study were analyzed and compared to those observed with the control strain (Figure S7). The strains producing the most PHB following induction with l-arabinose were PAN (19.80 g/L) and CBB_phaA (19.78 g/L), resulting in yields of up to 86.34 and 87.55% CDW, respectively. These values were slightly higher than those observed with the CTRL (18.5 g/L; 81.95%) and KI (18.30 g/L; 87.50%) strains. Despite yielding a maximum PHB content comparable to those observed with the above-mentioned strains (85.69%), CBB produced significantly less PHB following induction (10.38 g/L) than the other *C*. *necator* H16 derivatives. The most likely explanation for this result is that, at the time of induction, CBB presented significantly higher PHB content (∼72%), with respect to all of the other strains (PHB yields at time of induction ranging between ∼50 and ∼57% of CDW). These observations suggest that a significantly lower portion of the CO_2_ metabolized by CBB following l-arabinose induction was employed for PHB biosynthesis, with respect to the other strains, potentially enhancing carbon flux through the MVA pathway. Hence, the amounts of CO_2_ consumed by the *C*. *necator* H16 derivatives tested in this study during each of the 24 h postinduction time intervals were used to calculate their respective partial MVA yields. The overall MVA yields for each strain were obtained as the sum of their corresponding partial MVA yields. The data obtained are reported in Table S4. A summary of the pre- and postinduction gas consumption rates for each strain, along with their respective overall MVA yields, is shown in Table 1.

**Table 1:** Comparison between the Average CO_2_, H_2_, and O_2_ Uptake Rates (CUR, HUR, and OUR, Respectively) Measured Pre- and Post-Induction for Each of the H16 Derivatives Tested^a^

	CUR (mM/h)		HUR (mM/h)		OUR (mM/h)		
strain	PRE-induction	POST-induction	PRE-induction	POST-induction	PRE-induction	POST-induction	MVA yield (% C-mol)
CTRL	18.0	4.2	73.5	29.7	24.5	16.7	
PAN	35.5	6.7	270.0	93.2	63.0	29.5	10.8
CBB	35.3	6.5	230.6	80.5	52.5	27.3	25.0
KI	22.0	3.5	78.0	27.5	26.0	21.0	2.6
CBB-phaA	37.4	7.4	256.7	84.9	63.0	29.7	23.0
PHB-phaA	12.0	0.7	58.0	28.0	18.0	13.9	12.2

a Their respective MVA yields, calculated as the % of C-mol of CO_2_ consumed following induction, are also reported. Standard deviation of <0.05 was observed between replicates (regression analysis).

The data presented in Table 1 clearly show that strains CBB and CBB_phaA also outperformed PAN in terms of MVA production yields. Indeed, 25 and 23% of the total CO_2_ consumed by CBB and CBB_phaA after induction with l-arabinose was converted into MVA, while the overall MVA yield obtained with the PAN strain was only 10.8%. Moreover, MVA production by PAN stopped around the 144 h postinduction time point (Table S4), confirming that the *in trans* complementation of the Δ*panC* mutation is not the most effective way to improve plasmid stability in *C*. *necator* H16. The data presented in Table S4 also indicate that for all strains apart from CBB, the lowest MVA yields were obtained during the first 24 h following induction. This may be due to a lag in expression of the *mvaES* genes, encoding the enzymes responsible for MVA production or, most likely, because during the 0 to 24 h postinduction time interval, the strains were still actively duplicating, as indicated by the viable counts data reported in Figures 2B, 4A, and 6A. This suggests that a significant portion of the total CO_2_ consumed by each strain during this time may have been used to produce biomass and PHB. Cell maintenance and, in the case of PAN, pantothenate production are additional processes that required CO_2_. However, the main fermentation product was observed in all of the *C*. *necator* H16 derivatives tested here, apart from the ΔPHB_phaA strain, was PHB. It is also interesting to note that even though strains CBB and CBB_phaA showed comparable overall MVA yields, the latter strain consumed over 2-fold more CO_2_ than CBB, following induction of MVA biosynthesis (Tables S4 and S5). This is consistent with the observations that CBB_phaA produced ∼2-fold higher amounts of both MVA and PHB, with respect to CBB (Table S5). Therefore, it can be inferred that the overexpression of PhaA in the CBB strain genetic background seems to increase the intracellular pool of acetoacetyl-CoA significantly, boosting metabolic fluxes toward both MVA and PHB biosynthesis. Since MVA and PHB are both commercially valuable products, it can be speculated that employing CBB_phaA as microbial chassis to produce these chemicals stably and sustainably may improve the economics of the proposed gas fermentation process. However, it must be noted that PhaA overexpression also increases the demand for CO_2_ and H_2_, which, particularly the latter, can impact production costs significantly. The calculated theoretical maximum yields of MVA and PHB are 0.04 and 0.06 mol/mol of H_2_, respectively. The observed yield for the different strains based on H_2_ consumption and MVA production is between 0.002 and 0.004 mol MVA/mol H_2_, which only accounts for about 9% of the theoretical maximum yields. Therefore, a detailed technoeconomic analysis, which is beyond the scope of the present study, is required to investigate the feasibility of scaling up this dual-product fermentation process.

### 3.9 MVA Production in Other Biobased Fermentation Processes

Bioproduction of MVA has been previously attempted, using different combinations of microbial chassis and carbon sources, as summarized in Table 2. The highest MVA titers described so far were obtained from glucose. Despite being up to 7–8 times higher than the MVA concentrations described in this study, these titers are still far from being industrially valuable. Indeed, considering first or second-generation feedstock costs, MVA titers would need to be around 150–200 g/L,^63^ with volumetric productivities of no less than 2–3 g/L/h^64^ for commercial feasibility. It is also important to point out that fermentation processes employing organic feedstocks are not environmentally sustainable since a significant portion of the carbon metabolized by the microbial chassis is converted to the GHG CO_2_. The hereby conceptualized MVA production platform under autotrophic conditions addresses this drawback. However, the TYP metrics achieved in this study should also be increased to become industrially amenable based on current commercial models for bioproduction of chemicals from C1 waste gases (e.g. bioethanol production by Lanzatech Inc.). A techno-economical report investigating the viability of employing aerobic gas fermentation for producing bulk chemicals was published recently.^65^ In this study, isopropanol (IPA) production from waste gases (CO_2_ and H_2_) has been shown to be commercially viable if IPA was priced at 1000 $/kg, considering IPA titers of 12.4 g/L and productivity of 1.46 g/L/h in *C*. *necator*. Since MVA is of far greater value than IPA, with retail market prices of ∼10 $/mg (sources: Sigma-Aldrich Ltd., Biosynth Ltd.) and bulk market prices likely in the region of 100 $/g, the CBB_phaA strain described in this study constitutes a solid starting platform for further chassis development efforts, aiming toward commercially viable productivity targets (>1 g/L/h).

**Table 2:** Summary of MVA Production Levels Obtained from Various Fermentation Substrates with Different Microbial Chassis

strain	substrate	titers (g/L)	refs
*E*. *coli*	glucose	73.30	(66)
*Saccharomyces cerevisiae*	glucose	3.80	(66)
*Pseudomonas putida*	glycerol	0.24	(66)
*M*. *bacilli*	methanol	0.34	(66)
*C*. *necator*	CO_2_ + H_2_	10.00	this study

## 4 Conclusions

In conclusion, our data demonstrate that the RubisCO-based plasmid addiction system can be successfully employed to improve plasmid stability in *C*. *necator* H16, in laboratory-scale batch fermentation processes, carried out under autotrophic conditions. Importantly, this resulted in a significant increase in MVA production, obtaining titers of around 10 g/L, with an overall yield of ∼25% in terms of postinduction CO_2_ consumption. To the best of our knowledge, this is the first study reporting the production of “non-native” C6 molecules from C1 feedstocks in *C*. *necator* H16. This is a remarkable achievement considering that no MVA production could be observed in the control strain in the absence of plasmid addiction systems. In terms of gas utilization, the best MVA producer (strain CBB_phaA) consumed significantly more CO_2_, O_2_, and H_2_ than the CTRL strain. If in the case of the GHG CO_2_ this is not a critical issue and may even add value to the process, at least in terms of sustainability, the significantly higher amounts of H_2_ consumed by CBB_phaA, as compared to the control strain, may impact process economics, potentially hindering the application of the RubisCO-based plasmid addiction system to larger-scale fermentation processes. A possible way to address this problem may be the screening of a promoter library to fine-tune the expression of the plasmid-borne *cbbLS* genes, thus decreasing their metabolic burden on *C*. *necator* H16 cells and reducing gas consumption to levels comparable to those of the CTRL strain. A similar approach could be employed to optimize PhaA expression levels. Despite further adjustments, the RubisCO-based system represents the most successful attempt to improve the plasmid stability in autotrophically grown *C*. *necator* H16 that has been described to date. In addition, this plasmid addiction system has the potential to be implemented in other facultative chemolithoautotrophic bacterial species that use the CBB cycle as their sole or primary metabolic route for CO_2_ fixation.

## Supporting Information Available

Schematic diagram of the overall concept for the plasmid addition system and product synthesis; molecular cloning strategies to construct the plasmids used in this study; strains and plasmids used in this study; oligonucleotide primers used in this study; MVA production in *C*. *necator* H16 under heterotrophic conditions; shake flask cultivation experiments; SDS-PAGE analysis; complementation of the *C*. *necator* H16 Δ*panC* mutant with plasmid pMTL71301::panC; correlation between levels of “social cheaters” and MVA productivity in PAN strain; CO_2_, O_2_, and H_2_ gas consumption after induction with 0.2% l-arabinose in CTL and engineered strains; biomass production and PHB yields in CTL and engineered strains; and MVA yields per mmol of CO_2_ consumed by the CTL and engineered strains (PDF)
