# Supplementary material for: Stable Platform for Mevalonate Bioproduction from CO2
Source: ACS Sustain Chem Eng. 2024 Aug 26;12(36):13486–99. doi: 10.1021/acssuschemeng.4c03561 (PMC11388446; doi:10.1021/acssuschemeng.4c03561)
Supplement: Supplementary file 1 — sc4c03561_si_001.pdf [file sc4c03561_si_001.pdf]

SUPPORTING INFORMATION

Title: Stable platform for mevalonate bioproduction from CO_2

Authors: Marco Garavaglia[a], Callum McGregor[a b], Rajesh Reddy Bommareddy[a c], Victor Irorere[a d], Christian Arenas[a b], Alberto Robazza[a e], Nigel Peter Minton[a] and Katalin Kovacs[a f*]

[a] BBSRC/EPSRC Synthetic Biology Research Centre (SBRC), Biodiscovery Institute, School of Life Sciences, The University of Nottingham, Nottingham, NG7 2RD, UK

[b] Better Dairy Limited, Unit J/K Bagel Factory, 24 White Post Lane, London, E9 5SZ, UK

[c] Hub for Biotechnology in the Built Environment, Department of Applied Sciences, Faculty of Health and Life Sciences, Northumbria University, Ellison Building, Newcastle upon Tyne, NE1 8ST, UK

[d] DSM-Firmenich, 250 Plainsboro Road, Plainsboro, NJ, 08536, United States

[e] Karlsruhe Institute of Technology (KIT), PO Box 6980, Karlsruhe, 76049, Germany

[f] School of Pharmacy, University Park, The University of Nottingham, Nottingham, NG7 2RD, UK

[*] corresponding author: katalin.kovacs@nottingham.ac.uk

Number of pages: 32

Number of figures: 7

Number of tables: 5

Schematic diagram of the overall concept for the plasmid addition system and product synthesis from CO_2 in engineered *C. necator* H16 strains.

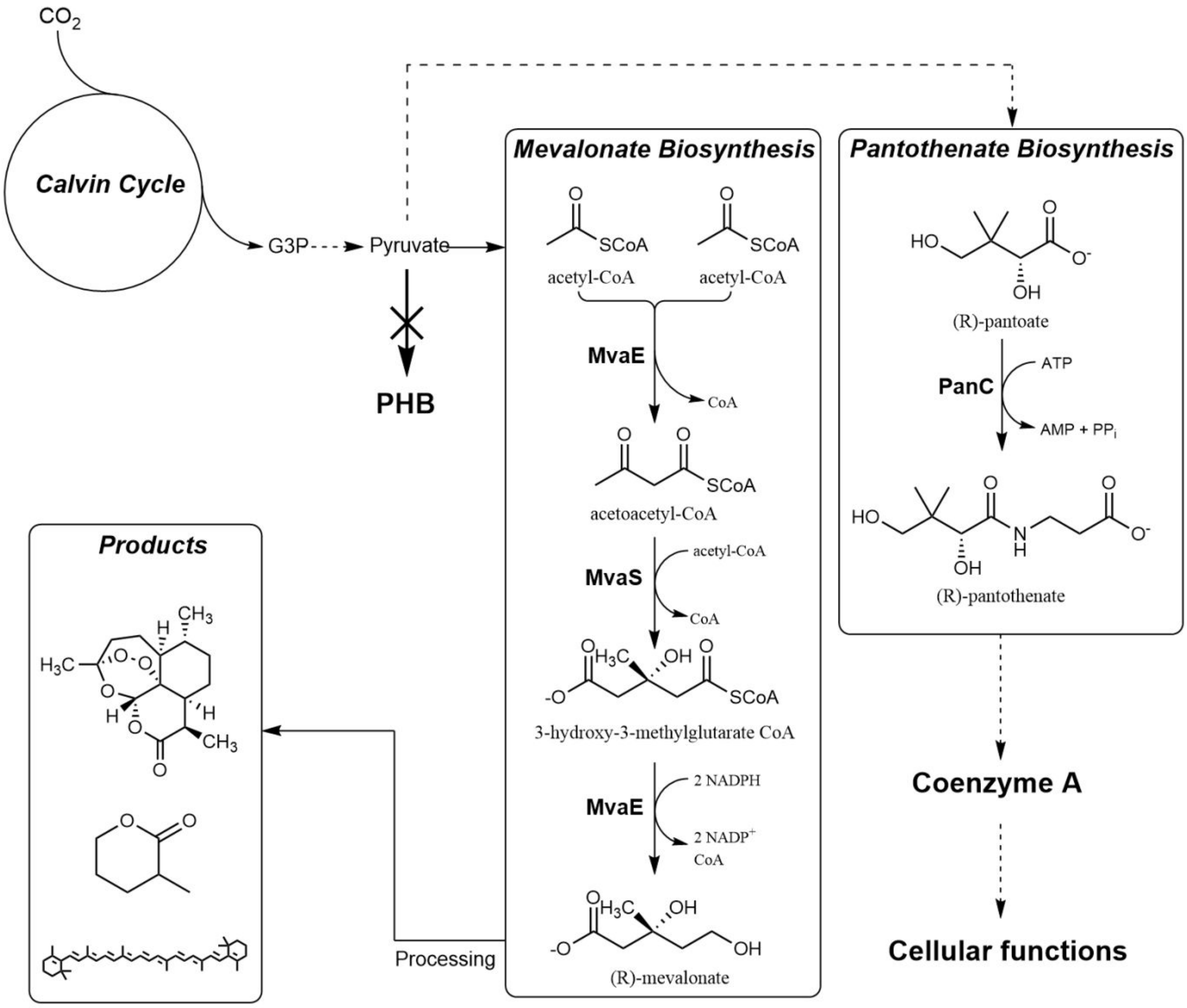

Figure S1 – Overview of pathways involved in mevalonate-producing strains of *C. necator H16*. CO_2 is converted to pyruvate by the reactions of the Calvin cycle and glycolysis. Pyruvate acts as the starting compound for PHB, mevalonate, and pantothenate biosynthesis. The PHB biosynthetic operon can be inactivated to redirect the carbon flux towards mevalonate synthesis. To facilitate retention of the mevalonate production pathway during continuous fermentation, the genomic copy of pantothenate synthetase (*panC*) or the large subunits of the **RuBisCO (*ΔΔcbbLS*)** were deleted. The *panC* gene was then placed, with its native promoter on the backbone of the plasmid carrying the mevalonate pathway. Similarly to the *panC* complementation, the large subunit of the RuBisCo gene was placed under a medium-strength promoter on the backbone of the plasmid carrying the mevalonate pathway. The addiction systems described above allowed for continuous production of mevalonate from CO_2. Dashed lines indicated multiple reactions. Acetyl-CoA acetyltransferase/HMG-CoA reductase (MvaE), HMG-CoA synthase (MvaS), pantothenate synthetase (PanC). Products from top to bottom: artemisinin (anti-malarial compound), β-methyl-Δ-valerolactone ((βMΔVL) precursor to biodegradable

polymer), and β-carotene (precursor to compounds relevant to the pharmaceutical, cosmetics, and food industries).

Molecular cloning strategies to construct the plasmids used in this study

The *panC* deletion plasmid pMTL70621::Δ*panC* was constructed by amplifying the 750 bp DNA regions located just upstream and downstream of the *panC* gene on *C. necator* H16 chromosome 1, using primer pairs panC_LHA_FW + panC_LHA_REV and panC_RHA_FW + panC_RHA_REV. The resulting fragments were assembled into a deletion cassette via size overlap extension PCR. Plasmid pMTL70621-SacB was digested with NotI and NdeI restriction enzymes and the deletion cassette ligated into the plasmid.

Plasmids pMTL70621::Δ*cbbLS*2 and pMTL70621::Δ*cbbLS*p, respectively used to delete the *cbbLS*2 and *cbbLS*p genes, were constructed by amplifying the circa 1000 bp DNA regions located just upstream and downstream of the *cbbLS*2 operon on *C. necator* H16 chromosome 2 and of the *cbbLS*p operon on the pHG1 megaplasmid. In the case of Δ*cbbLS*2, the upstream and downstream homology arms were amplified using primer pairs LHA_cbbLSch_FW + LHA_cbbLSch_RV and RHA_cbbLSch_FW + RHA_cbbLSch_RV, respectively. Primer pairs LHA_cbbLSm_FW + LHA_cbbLSm_RV and RHA_cbbLSm_FW + RHA_cbbLSm_RV were instead used to obtain the left and right homology arms for *cbbLS*p. The deletion cassettes were then cloned in the XbaI/NcoI-digested pMTL70621-SacB vector, using the NEBuilder® HiFi DNA assembly method, to obtain pMTL70621::Δ*cbbLS*2 and pMTL70621::Δ*cbbLS*p. The *panC* complementation plasmid pMTL71301::*panC* was obtained by cloning the *panC* gene from *C. necator* H16, including its native promoter and Rho-independent terminator of transcription, in the AscI/FseI-digested modular shuttle vector

pMTL71301. The P_{panC}-*panC*-T_{panC} DNA construct was amplified from *C. necator* H16 chromosome 1, using primers panC_FW_HiFi and panC_REV_HiFi.

The *cbbLS* complementation plasmid pMTL71301::T500-P_{phaC}-*cbbLS*2 was constructed by amplifying the promoter of the *phaCAB* operon from *C. necator* H16 chromosome 1, using primers T_PphaC_fwd (which contains the T500 synthetic terminator sequence) and PphaC_rev, and the *cbbLS*2 operon from chromosome 2, using the primers cbbLS_fwd and cbbLS_rev. The T500- P_{phaC} and *cbbLS*2 DNA parts were then cloned in the XbaI/NheI-digested pMTL71301 vector, using the NEBuilder® HiFi DNA assembly method.

To construct the MVA producing plasmids pMTL71301::*araC*-P_{BAD}-*mvaES* and pMTL71301::*araC*-P_{BAD}-*mvaES*::*panC*, the three DNA parts carrying the *araC*-P_{BAD} promoter cassette, the *E. coli* codon-optimised versions of the *Enterococcus faecalis mvaE* and *mvaS* genes were PCR-amplified, using the pBbA1kMVALS plasmid (a kind donation by SYNBIOCHEM, Manchester) as the DNA template and the primer pairs ara_fwd / ara_rev; mvaE_fwd / mvaE_rev and mvaS_fwd in combination with either mvaS_rev or mvaS_REV2, for pMTL71301::*araC*-P_{BAD}-*mvaES*::*panC* and pMTL71301::*araC*-P_{BAD}-*mvaES,* respectively. The amplified DNA parts were then cloned in either the pMTL71301 or pMTL71301::*panC* plasmids (NotI/XbaI-digested), using the NEBuilder® HiFi DNA assembly method.

To construct plasmid pMTL70621::KI_A3739::*araC*-P_{BAD}-*mvaES::*A3740, the circa 780bp DNA sequences encompassing the intergenic regions found between the H16_A3739 and H16_A3740 genes were amplified using primer pairs 3739INT3740_UP_FWD + 3739INT3740_UP_REV and 3739INT3740_DOWN_FWD + 3739INT3740_DOWN_REV. The UP and DOWN homology arms were then cloned

in the NotI/XhoI-digested pMTL70621-SacB vector, using the NEBuilder® HiFi DNA assembly method, resulting in plasmid pMTL70621::KI_A3739::INT::A3740. To allow cloning of the *araC*-P_{BAD}-*mvaES* operon in between the homology arms, a cutting site for XbaI (provided as a spacer in the 3739INT3740_UP_REV primer sequence) was introduced downstream of the UP sequence. Primer pairs IR_ara_FWD + A3743_ara_REV, A3743_mvaE_FWD + A3743_mvaE_REV and A3743_mvaS_T500_FWD + IR_mvaS_T500_REV were used to amplify the *araC*-P_{BAD} promoter cassette, the *mvaE* and *mvaS* genes, respectively. Plasmid pMTL71301::*araC*-P_{BAD}-*mvaES* was used as a template for these PCRs. The synthetic Rho-independent terminator T500 was introduced downstream of *mvaS* by including it in the IR_mvaS_T500_REV primer sequence. These DNA parts were then cloned together in the XbaI-digested pMTL70621::KI_A3739::INT::A3740, using NEBuilder® HiFi, to obtain plasmid pMTL70621::KI_A3739::*araC*-P_{BAD}-mvaES::A3740, which was used to integrate the MVA pathway into *C. necator* chromosome 1.

The pMTL71301::*araC*-P_{BAD}-*mvaES*-P_{phaC}-*cbbLS* plasmid was constructed by amplifying the T500-P_{phaC}-*cbbLS*2 DNA construct, using primers mvaS-T-PphaC_fwd and cbbLS_rev. This was then coned in the XbaI/NheI-digested pMTL71301::*araC*-P_{BAD}-*mvaES* plasmid, using the NEBuilder® HiFi DNA assembly method.

The pMTL71301::*araC*-P_{BAD}-*phaAmvaES*-P_{phaC}-*cbbLS* plasmid was constructed by amplifying the *araC*-P_{BAD} promoter cassette and the *mvaES* operon from plasmid pMTL71301::*araC*-P_{BAD}-*mvaES*, using primer pairs ara_fwd / Pbad_rev and mvaES_fwd / mvaS_REV2, while the *phaA* gene, alongside with its native RBS, was amplified from *C. necator* H16 chromosome 1 with primers phaA_fwd and phaA_rev. The *araC*-P_{BAD}, *phaA* and *mvaES* parts were then cloned in the NotI/XbaI-digested

pMTL71301::T500-P_{phaC}-*cbbLS*2 plasmid, using the NEBuilder® HiFi DNA assembly method.

Table S1. Strains and plasmids used in this study.

**Strains or plasmids**	**Description**	**Reference or Source**
Bacterial Strain		
Cupriavidus necator		
H16	Wild-type strain; Gm^r	ATCC17699
Δ*panC*	Mutant of H16 with deleted *panC* gene	This study
ΔΔ*cbbLS*	Mutant of H16 with deleted *cbbLS*2 and *cbbLS*p operons	This study
KI_A3739::*araC*-P_{BAD}-*mvaES*::A3740	H16 derivative carrying the MVA synthetic pathway integrated on chromosome 1, at the level of the intergenic region between H16_A3739 and H16_A3740	This study
ΔΔ*cbbLS*, Δ*phaCAB*	Mutant of H16 with deleted *cbbLS*2, *cbbLS*p and *phaCAB* operons	This study
Escherichia coli		
DH5α	Strain for plasmid cloning and propagation	Invitrogen
S17-1	Strain for conjugative transfer of plasmids to *C. necator* H16	Invitrogen
Plasmids		
pMTL71301	Base vector for cloning and expression of genes; Tet^R	(Ehsaan et al., 2021)
pMTL71301::*araC*-P_{BAD}-*mvaES*	pMTL71301 expressing *mvaE* and *mvaS* from *Enterococcus faecalis*	This study

pMTL71301::*panC*	pMTL71301 expressing *panC* from *C.necator* H16	This study
pMTL71301::*araC*-P_{BAD}-*mvaES:: panC*	pMTL71301 expressing *panC* from *C. necator* H16, *mvaE* and *mvaS* from *Enterococcus faecalis*	This study
pMTL71301::P_{phaC}-*cbbLS2*	pMTL71301 expressing *cbbLS2* from *C.necator* H16	This study
pMTL71301::*araC*-P_{BAD}-*mvaES::cbbLS2*	pMTL71301 expressing *cbbLS2* from *C.necator* H16, *mvaE* and *mvaS* from *Enterococcus faecalis*	This study
pMTL71301::*araC*-P_{BAD}-*phaA*-*mvaES::cbbLS2*	pMTL71301 expressing *cbbLS2* and *phaA* from *C.necator* H16, *mvaE* and *mvaS* from *Enterococcus faecalis*	This study
pMTL70621-SacB	Base vector for gene deletion and integration; carries *sacB,* Tet^R	(Ehsaan et al., 2021)
pMTL70621::*ΔpanC*	70621 carrying deletion cassette for *panC* gene	This study
pMTL70621::*ΔcbbLS2*	70621 carrying deletion cassette for *cbbLS2* operon (on chromosome 2)	This study
pMTL70621::*ΔcbbLSp*	70621 carrying deletion cassette for *cbbLSp* operon (on pHG1 megaplasmid)	This study
pMTL70621::*ΔphaCAB*	70621 carrying deletion cassette for *phaCAB* operon	This study
pMTL70621::KI_A3739::*araC*/P_{BAD}:: *mvaES*::A3740	70621 carrying the *araC*/P_{BAD}::*mvaES* synthetic operon located within the integration cassette targeting the intergenic region between H16_A3739 and H16_A3740	This study

Table S2. Oligonucleotide primers used in this study.

Primer	5’ to 3’ sequence	Details
panC_LHA_FW	ATGGCGGCCATGCTGATCGAGATCAAG	Primers used to amplify the DNA sequence upstream of the *panC* gene in the *C. necator* H16 genome to obtain the left homology arm for constructing the *panC* in-frame deletion
panC_LHA_REV	CATCTGCCTTGGTGTGCTGGTCG	
panC_RHA_FW	TGAGCGGCAGCGCCGCACCAGGC	Primers used to amplify the DNA sequence downstream of the *panC* gene in the *C. necator* H16 genome to obtain the right homology arm for constructing the *panC* in-frame deletion
panC_RHA_REV	TGTTGCTGACCACGCTGGTGGGCGTG	
LHA_cbbLSch_FW	gctcggtacccggggatcctctagaGTGCGCAGCTCCAGTCCCAGCGTG	Primers used to amplify the DNA sequence upstream of the *cbbLS2* operon on *C. necator* H16 chromosome 2 to obtain the left homology arm for constructing the *cbbLS2* in-frame deletion
LHA_cbbLSch_RV	tcgagcatccGCTTGTCTCCTTGCGTGGTTGAGCG	
RHA_cbbLSch_FW	ggagacaagcGGATGCTCGATTGATCCTCACGGAGCC	Primers used to amplify the DNA sequence downstream of the *cbbLS2* operon on *C. necator* H16 chromosome 2 to obtain the right homology arm for constructing the *cbbLS2* in-frame deletion
RHA_cbbLSch_RV	tctgcaggcctcgagatctccatggACACGCGGCTGGCAAGCAGGTCAG	
LHA_cbbLSm_FW	gctcggtacccggggatcctctagaGCGGTTGCCTTCGGCGAAGCGGA	Primers used to amplify the DNA sequence upstream of the *cbbLSp* operon on *C. necator* H16 pHG1 megaplasmid to obtain the left homology arm for constructing the *cbbLSp* in-frame deletion
LHA_cbbLSm_RV	ccggtgccgcGCTTGTCTCCTTGCGTGGTTGAGCGTC	
RHA_cbbLSm_FW	ggagacaagcGCGGCACCGGCTGAACCGCCGCGC	Primers used to amplify the DNA sequence downstream of the *cbbLSp* operon on *C. necator* H16 pHG1 megaplasmid to obtain the right homology arm for constructing the *cbbLSp* in-frame deletion
RHA_cbbLSm_RV	tctgcaggcctcgagatctccatggCCCAAGGCCTTCTTCAGGATCTCCTTGGTCTTGGGCGC	
panC_FW_HiFi	Caggcttcttatttttatggcgcgcccccacccgcgctgcgggg	Primers used to amplify the P_{panC}-*panC*-T_{panC} DNA construct from *C. necator* H16 chromosome 1
panC_REV_HiFi	tctggcgctcctgcggccggccaaaaagcaggccgccggcctgc	
T_PphaC_fwd	acgaattcgagctcggtacccggggatcctctagacaaagcccgccgaaaggcgggcttttctgtCGCTCATCCTTCTCGCCTATG	Primers used to amplify the P_{phaC} promoter from *C. necator* H16 chromosome 1. Primer T_PphaC_fwd also includes the synthetic Rho-independent terminator T500 (underlined)
PphaC_rev	tcaggtgcgttcataTGTTGATTGTCTCTCTGCCG	
cbbLS_fwd	gagagacaatcaacaTATGAACGCACCTGAATCGGTC	Primers used to amplify the *cbbLS2* operon from *C. necator* H16 chromosome 2. Primers pair T_PphaC_fwd + cbbLS_rev was used to amplify the T500-P_{phaC}-*cbbLS2* DNA construct to obtain plasmid pMTL71301::*araC*-P_{BAD}-*mvaES*-P_{phaC}-*cbbLS*
cbbLS_rev	caaatgcaggcttcttatttttatgctagcGTACCTCAGTAGCGGCT	

ara_fwd	ctacaatttttttatcaggaaacagctatgaccgcggccgcTTAAGCAGAAGGCCATCC	Primers used to amplify the *araC*/P_{BAD} L-arabinose-inducible promoter cassette from plasmid pMTL71102 (Ehsaan et al., 2021)
ara_rev	aatcaccacggttttcatATGTATATCTCCTTCTTAAAAGATCTTTTG	
mvaE_fwd	taagaaggagatatacatATGAAAACCGTGGTGATTATTG	Primers used to amplify the *E. faecalis mvaE* gene from plasmid pBbA1kMVALS
mvaE_rev	ttattttgtcgctaatcaTTACTGTTTACGCAGATCATTC	
mvaS_fwd	gatctgcgtaaacagtaaTGATTAGCGACAAAATAAATAAC	Primers used to amplify the *E. faecalis mvaS* gene from plasmid pBbA1kMVALS. Primer mvaS_fwd was used in combination with either mvaS_rev or mvaS_REV2 to amplify the DNA parts carrying *mvaS* that were used to construct plasmids pMTL71301::*araC*-P_{BAD}-*mvaES::panC* and pMTL71301::*araC*-P_{BAD}-*mvaES*, respectively
mvaS_rev	tatttttatgctagttgatcagttatctagatccggtggatccTTAATTACGATAGCTACGC	
mvaS_REV2	gagatctccatggacgcgtgacgtcgactctagaggatccTTAATTACGATAGCTACGC	
3739INT3740_UP_FWD	tttatcaggaaacagctatgaccgcggccgcATCCGCTATGAGGTGC	Primers used to amplify the DNA region encompassing the final portion of the H16_A3739 gene and part of the intergenic region between genes H16_A3739 and H16_A3740. This was used as the left homology arm for facilitating the integration of *araC*-P_{BAD}-*mvaES*-T500 in *C. necator* H16 chromosome 1
3739INT3740_UP_REV	gattcggtcgttctagaGCGTTTGAAACCGGGAG	
3739INT3740_DOWN_FWD	cggtttcaaacgctctagaACGACCGAATCCCATGG	Primers used to amplify the DNA region encompassing part of the intergenic region between genes H16_A3739 and H16_A3740 and the initial portion of the H16_A3740 gene. This was used as the right homology arm for facilitating the integration of *araC*-P_{BAD}-*mvaES*-T500 in *C. necator* H16 chromosome 1
3739INT3740_DOWN_REV	tgccaagcttgcatgtctgcaggcctcgagACTGGATCGCGCTGGC	
IR_ara_FWD	cgaagcctgaaaaaaatctcccggtttcaaacgctctagaTTAAGCAGAAGGCCATCC	Primers used to amplify the *araC*-P_{BAD} promoter cassette for assembly of plasmid pMTL70621::KI_A3739::*araC*/P_{BAD}::*mvaES*::A3740
A3743_ara_REV	aatcaccacggttttcatATGTATATCTCCTTCTTAAAAGATCTTTTG	
A3743_mvaE_FWD	taagaaggagatatacatATGAAAACCGTGGTGATTATTG	Primers used to amplify the *mvaE* gene for assembly of plasmid pMTL70621::KI_A3739::*araC*/P_{BAD}::*mvaES*::A3740
A3743_mvaE_REV	ttattttgtcgctaatcaTTACTGTTTACGCAGATCATTC	

A3743_mvaS_T500_FWD	gatctgcgtaaacagtaaTGATTAGCGACAAAATAAATAAC	Primers used to amplify the *mvaS* gene for assembly of plasmid pMTL70621::KI_A3739::*araC*/P_{BAD}::*mvaES*::A3740. Primer IR_mvaS_T500_REV also includes the synthetic Rho-independent terminator T500 (underlined)
IR_mvaS_T500_REV	ccaccccgcaggcgcttgcgccatgggattcggtcgttctagacaaagcccgccgaaaggcgggcttttctgtTTAATTACGATAGCTACGC	
Pbad_rev	ccggcactcatgcaagcgATGGAGAAACAGTAGAGAG	Primer used in combination with ara_FWD to amplify the *araC*- P_{BAD} promoter cassette for assembly of plasmid pMTL71301::*araC*-P_{BAD}-*phaA-mvaES*::*cbbLS2*
mvaES_fwd	gcagtcgagcgcaaataaGAATTCAAAAGATCTTTTAAGAAGG	Primers used to amplify the *mvaES* operon for assembly of plasmid pMTL71301::*araC*-P_{BAD}-*phaA-mvaES*::*cbbLS2*
mvaES_rev	agcgacagaaaagcccgcctttcggcgggctttgtctagaTTAATTACGATAGCTACGCACGGTGTTATTAATG	
phaA_fwd	tctctactgtttctccatCGCTTGCATGAGTGCCGG	Primers used to amplify the *phaA* gene from *C. necator* H16 genome for assembly of plasmid pMTL71301::*araC*-P_{BAD}-*phaA-mvaES*::*cbbLS2*
phaA_rev	cttaaaagatcttttgaattcTTATTTGCGCTCGACTGCC	
panC_EXT_FW	CCGCAGGACCTGTACATCC	Primers annealing on *C. necator* H16 genome, just outside of the left and right homology arms used to create the *panC* in-frame deletion. These were used to screen for mutants carrying the deletion of the DNA sequence of interest
panC_EXT_REV	GTATATCGGCCTGAGCCTTG	
panC_INT_FW	CAACTGCGCGGCCAGAAC	Primers annealing on *C. necator* H16 genome, within the *panC* gene. These were used to confirm the absence of the wild type allele of *panC* in the *ΔpanC* mutants
panC_INT_REV	CCAGCTTGGCGGCAGTCAG	
cbbLSch_OUT_FW	GCAGTGTGCTCCAGCAAATAGGC	Primers annealing on *C. necator* H16 chromosome 2, just outside of the left and right homology arms used to create the *cbbLS2* in-frame deletion. These were used to screen for mutants carrying the deletion of the DNA sequence of interest
cbbLSch_OUT_RV	GCTTGCATGGCTTACTCCTTGGC	
cbbLSch_IN_FW	CGTTCCGCGTGATTGCCAAATGG	Primers annealing on *C. necator* H16 chromosome 2, within the *cbbLS2* operon. These were used to confirm the absence of functional *cbbLS2* genes in the *ΔcbbLS2* and *ΔΔcbbLS* mutants
cbbLSch_IN_RV	CGGATTGCACAGTCAGGAAATCG	
cbbLSm_OUT_FW	GAAGGTTTCGTGGCGTAGTTCTTG	Primers annealing on *C. necator* H16 pHG1 megaplasmid, just outside of the left and right homology arms used to create the *cbbLSp* in-frame deletion. These were used to screen for mutants carrying the deletion of the DNA sequence of interest
cbbLSm_OUT_RV	GTAGTAGGCCTCGTCGATGAAGAGC	
cbbLSm_IN_FW	CAGAACGACATGATCCTGCACCTGC	Primers annealing on *C. necator* H16 pHG1 megaplasmid, within the *cbbLSp* operon. These were used

cbbLSm_IN_RV	GTTCAGGCAGTATTCAAGCTGGCTG	to confirm the absence of functional *cbbLSp* genes in the *ΔΔcbbLS* mutants
A3739_EXT_FOR<br><br>JW5seq3_REV2	GTCTCGCTCGCCTACCTC<br><br>GCCTGCAGAACATTGCCAAA	Primers used to screen for *C. necator* H16 mutants harbouring the genomic integration of *araC*-P_{BAD}-*mvaES*-T500. Primer A3739_EXT_FOR anneals on chromosome 1, upstream of the left homology arm carried by plasmid pMTL70621::KI_A3739::*araC*/P_{BAD}::*mvaES*::A3740. Primer JW5seq3_REV2 anneals within the *araC*-P_{BAD}-*mvaES*-T500 construct sequence.
JW5seq6_FOR<br><br>A3740_EXT_REV	GTTCTGGCAAGCCGTTATGC<br><br>GCGATTCCAGCATCCAGTAG	Primers used to screen for *C. necator* H16 mutants harbouring the genomic integration of *araC*-P_{BAD}-*mvaES*-T500. Primer JW5seq6_FOR anneals within the *araC*-P_{BAD}-*mvaES*-T500 construct sequence. Primer A3740_EXT_REV anneals on chromosome 1, downstream of the right homology arm carried by plasmid pMTL70621::KI_A3739::*araC*/P_{BAD}::*mvaES*::A3740.

MVA production in *C. necator* H16 under heterotrophic conditions

MVA is obtained from the condensation of three Acetyl-CoA molecules by the action of the acetyl-CoA acetyltransferase/HMG-CoA reductase (MvaE) and HMG-CoA synthase (MvaS) enzymes (Katsuki and Bloch, 1967). A schematic representation of the upper part of the MVA biosynthetic pathway is shown in Figure 1A. In this study, the MVA biosynthetic genes *mvaE* and *mvaS* from *Enterococcus faecalis* (*Ef-mvaE* and *Ef-mvaS*) were selected since these have been successfully employed to produce MVA and terpenoids in different bacterial species, including *Escherichia coli* and *Methylobacterium extorquens* (Tabata and Hashimoto, 2004; Wang et al., 2010; Yoon et al., 2009; Zhu et al., 2016). In particular, the *E. coli* codon-optimised versions of the *Ef-mvaE* and *Ef-mvaS* genes were cloned in the pMTL71301 modular shuttle vector

(Ehsaan et al., 2021), under the control of the *araC*/P_{BAD} L-arabinose-inducible promoter cassette, to obtain plasmid pMTL71301::*araC*-P_{BAD}-*mvaES*. This plasmid was transferred to *C. necator* H16 to obtain the *C. necator* H16/pMTL71301::*araC*-P_{BAD}-*mvaES* strain, which was then cultivated in nitrogen-limited MM, supplemented with 2.5% fructose and 10 μg/mL tetracycline. Ability to produce MVA under heterotrophic conditions was assessed, using the method described in the following paragraph. A *C. necator* H16 derivative carrying the empty pMTL71301 plasmid was used as the negative control strain in these experiments. To test whether MvaE and MvaS were being expressed correctly, samples were collected from the *C. necator* H16/pMTL71301 and *C. necator* H16/pMTL71301::*araC*-P_{BAD}-*mvaES* cultures right before providing the inducer L-arabinose into the media and after 24 h of induction. Whole-cell protein extracts were prepared from these culture samples and analysed by SDS-PAGE, as shown in Figure S1A. Significant amounts of the MvaE and MvaS proteins were detected in the H16/pMTL71301::*araC*-P_{BAD}-*mvaES* strain after 24h of induction with L-arabinose (Figure S1A). MvaE expression levels were significantly higher with respect to MvaS. This is likely because this protein is encoded by the first gene of the MVA biosynthetic operon *mvaES*. No expression of these proteins could be observed before adding the inducer L-arabinose into the culture media, suggesting that transcription of the *mvaES* operon is tightly regulated by the *araC*/P_{BAD} promoter cassette, despite the presence of multiple copies of this synthetic DNA construct per cell. In line with the protein expression data, the *C. necator* H16/pMTL71301::*araC*-P_{BAD}-*mvaES* strain also produced MVA under heterotrophic conditions, with a maximum MVA titre of around 2.4 g/L that was measured at the 48 h post-induction time point (Figure S1B). The concentration of MVA in the culture media did not vary significantly following this time point, suggesting that MVA production by *C. necator*

H16/pMTL71301::*araC*-P_{BAD}-*mvaES* might have stopped after 48 h of induction. The concentrations of MVA produced over time by *C. necator* H16/pMTL71301 and *C. necator* H16/pMTL71301::*araC*-P_{BAD}-*mvaES* strains, under these experimental conditions, were quantified by HPLC analysis (Figure S1). As expected, neither expression of MvaE and MvaS or MVA production could be observed in the negative control strain carrying the empty pMTL71301 vector.

Shake flask cultivation experiments

Single colonies of *C. necator* strains H16/pMTL71301 and H16/pMTL71301::*araC*-P_{BAD}-*mvaES* were used to inoculate 5 ml of LB liquid medium supplemented with 15 µg/mL tetracycline (in 50 mL Falcon tubes) and grown overnight at 30°C with shaking (200 rpm). After overnight incubation, the optical density (OD_{600}) of the cultures was measured and normalised to OD_{600}=0.1 in 100 mL of LB medium supplemented with 15 µg/mL tetracycline (in 500 mL flasks). The bacterial cultures were then incubated at 30 °C with shaking (200 rpm) until they reached an OD_{600} of around 0.6 - 0.7. At this point, 1 mL samples were collected from each culture, centrifuged at 14000 rpm for 1 minute and the cell pellets were stored at -20°C to be used for the purification of pre-induction protein samples for SDS-PAGE. In addition, 1 mL of 20% (w/v) *L*-arabinose solution was added to the cultures, thus obtaining a final *L*-arabinose concentration of 0.2%. Following induction with L-arabinose the bacterial cultures were incubated overnight at 30°C, with shaking. The following morning, the OD_{600} of the cultures was measured and 1 mL samples were removed from each of them to obtain the post-induction protein samples for SDS-PAGE. At this point, cells were harvested by centrifuging the cultures at 8000 rpm for 5 minutes. The pellets were then

resuspended in suitable volumes of 2.5% (w/v) Fructose Nitrogen-limited MM, supplemented with 15 µg/mL tetracycline, to obtain cultures with an OD_{600} of around 15. These were then transferred to 250 mL flasks and incubated at 30°C, with shaking. Culture samples (1mL at each time point) were then collected at 0, 1, 2, 3, 4, 5, 24, 48, 72, 96, 120, 144 and 168 hours from re-inoculation. These were centrifuged at 14000 rpm for 1 minute and the supernatants were filtered and prepared for HPLC analysis.

Preparation of protein samples for SDS-PAGE analysis

The pre- and post-induction cell pellets prepared as described in paragraph 2.6 were lysed using the BugBuster® protein extraction reagent (Merck-Millipore). The protein extraction mix was prepared by diluting BugBuster® (1:10); Protease Inhibitor Cocktail Set VII (1:50); rLysozyme™ (1:1000) and benzonase (1:10000) in appropriate volumes of PBS (all these reagents were purchased from Merck-Millipore). The total protein concentration in each sample was normalised using the formula: $[(OD_{600} / 0.2) * 45] / 2$. This was used to calculate the volume (in µL) of protein extraction mix to be added to each cell pellet. Cell pellets were then incubated at room temperature for 20 min, with gentle shaking (50 rpm). Resuspended cell pellets were then centrifuged at 14000 rpm for 10 min. 10 µL of the resulting supernatants (soluble protein fraction) were then mixed with 10 µL NuPAGE™ LDS sample buffer (Thermo Fisher Scientific) and boiled at 100ºC for 5 min. SDS-PAGE of protein samples was performed using NuPAGE™ Bis-Tris 4-12 % precast polyacrylamide gels (Thermo Fisher Scientific). Protein bands were visualised using GelCode™ Blue Safe Protein Stain (Thermo Fisher Scientific).

A

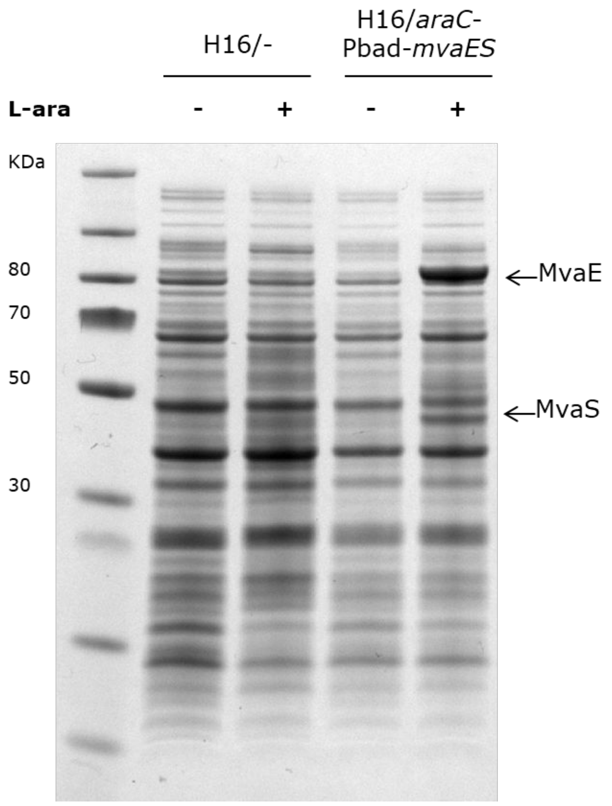

B

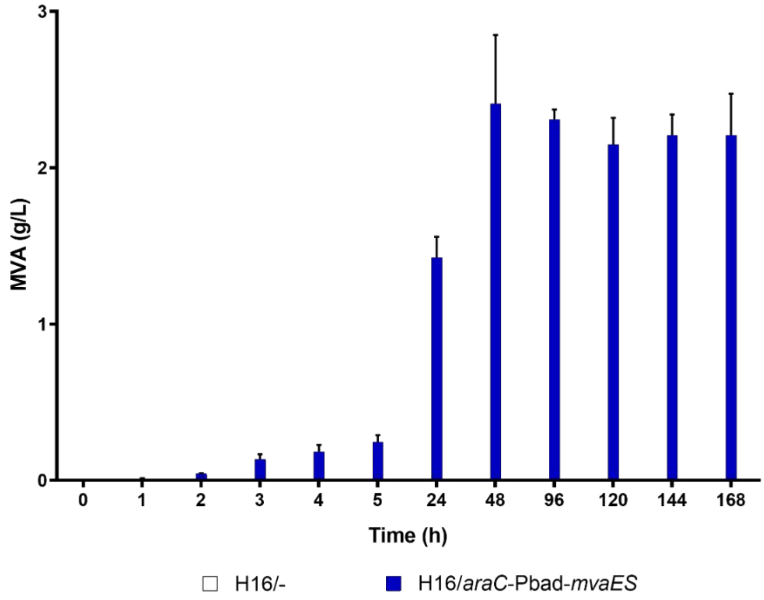

Figure S1. MvaE and MvaS expression and MVA production from fructose in *C. necator* H16/pMTL71301 and *C. necator* H16/pMTL71301::*araC*-P_{BAD}-*mvaES* (CTRL).

A) SDS-PAGE analysis of protein samples extracted from *C. necator* H16/pMTL71301 (H16/-) and *C. necator* H16/pMTL71301::*araC*-P_{BAD}-*mvaES* (CTRL). The – and + signs respectively indicate protein samples collected before and after inducing the cultures with 0.2% (w/v) L-arabinose. **B)** MVA titres (in g/L) produced by *C. necator* H16/pMTL71301 (white bars) and CTRL (dark blue bars) at the time of induction (t=0 h) and after 1, 2, 3, 4, 5, 24, 48, 72, 96, 120, 144 and 168 h of induction with 0.2% (w/v) L-arabinose in nitrogen-limited MM supplemented with 2.5% (w/v) fructose and 10 µg/mL tetracycline.

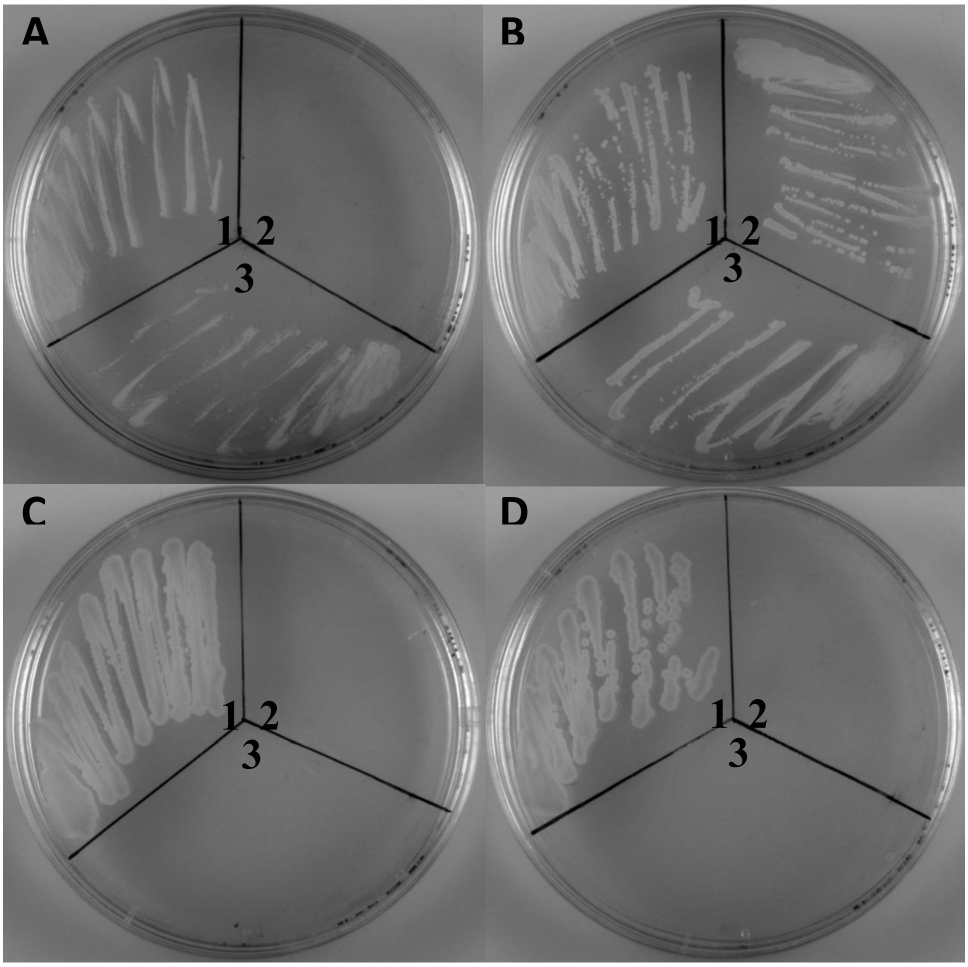

Figure S3. Complementation of the *C. necator* H16 *ΔpanC* mutant with plasmid pMTL71301::*panC*.

Complementation was shown to restore growth of the *C. necator* H16 *ΔpanC* mutant on media lacking pantothenate. **A)** SG-MM + 10 µg/mL Gm, no pantothenate added. **B)** SG-MM + 10 µg/mL Gm, 1 mM pantothenate. **C)** SG-MM + 10 µg/mL and 15 µg/mL Tet, no pantothenate added. **D)** SG-MM + 10 µg/mL and 15 µg/mL Tet, 1mM pantothenate. 1 = *C. necator* H16 *ΔpanC*/pMTL71301::*panC;* 2 = *C. necator* H16 *ΔpanC* and 3 = *C. necator* H16.

Correlation between levels of "social cheaters" and MVA productivity in PAN

A clear correlation was observed between the levels of social cheaters present in the PAN population and MVA production rates. To estimate the amounts of social cheaters, we firstly quantified the effects of pantothenate supplementation on the viable counts of the control strain (CTRL), by calculating the average increase in the number of cfu/mL observed on Pan, with respect to LB. This amount (15.87%) was then subtracted from the percentage of tetracycline sensitive (100 - % Tet-resistant cells) PAN cells at each time point to estimate the percentage of social cheaters (Figure S4). The partial MVA production rates, associated with each of the post-induction 24 h time intervals in between culture samples withdrawals, were determined by dividing the increments in MVA concentrations observed between two consecutive time intervals (ΔMVA) by 24 h (Figure S4).

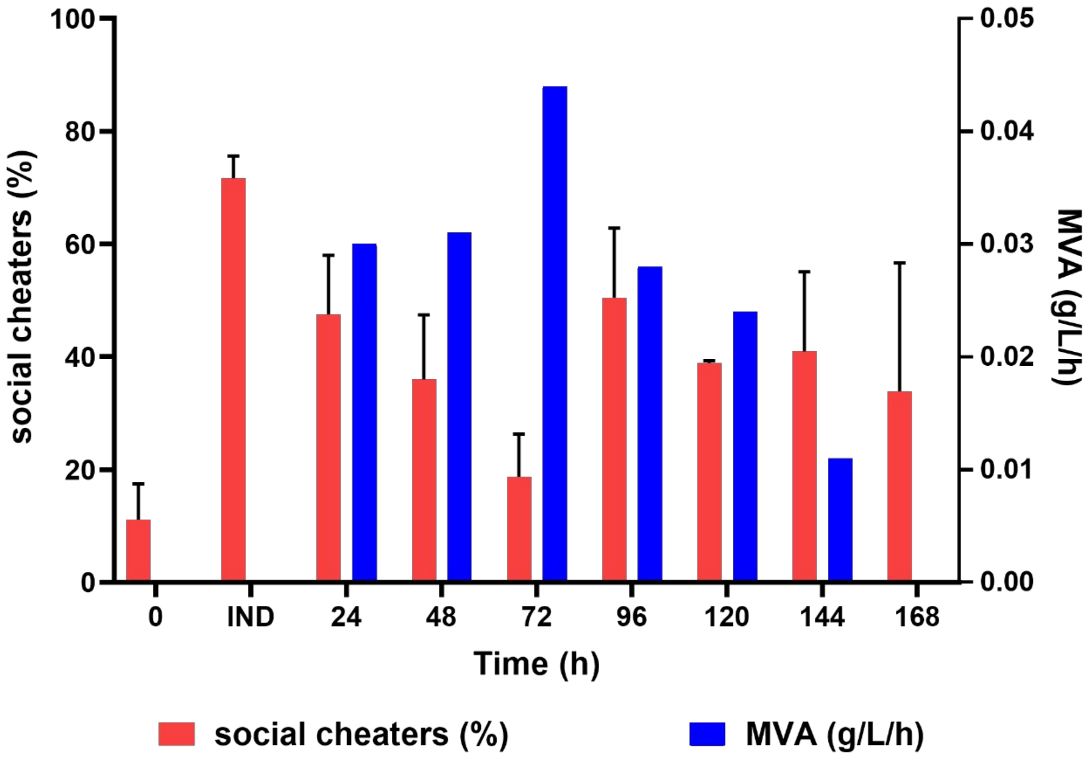

Figure S4. Correlation between percentage of social cheaters in the PAN population and MVA productivity.

The percentage of social cheaters (red bars) generated over time in the PAN population was calculated using the following formula: [100 - (cfu/mL Tet / cfu/mL Pan)] - 15.87, where the term between square brackets indicate the percentage of tetracycline-sensitive PAN cells at each time point and 15.87 is the average overall difference (in %) between the number of cfu/mL yielded by the CTRL strain on Pan, with respect to LB. Partial MVA production rates (g/L/h; blue bars) were obtained using the formula: ΔMVA (g/L) / 24, where ΔMVA is the difference in MVA concentrations (in g/L) observed between two consecutive culture samples collection time points.

As can be observed in Figure S4, the percentage of social cheaters in the PAN population increased significantly from the initial value determined at the time of inoculation (around 11%) to over 70% at the time of induction with 0.2% L-arabinose. This was the highest level of cheaters observed throughout the fermentation experiment, suggesting that higher percentage of cheaters may lead to population collapse. Indeed, under these conditions, the concentration of pantothenate present in the media is likely to be too low to sustain growth of such a large population of cheaters. As a result of this, during the first day of induction the average amounts of cheaters decreased by ~24% and the cooperators, which now represented just over half of the total PAN population, started producing MVA, with a productivity of around 0.030 g/L/h. MVA productivity (0.031 g/L/h) remained more or less constant during the second day of induction, at the end of which a further reduction (-11.61%) of the cheaters levels was observed. It is important to note that the highest MVA productivity (0.044 g/L/h) for this fermentation process was observed between the 48 h and 72 h post-induction time points, precisely the time interval when the portion of cheaters reached the lowest level observed throughout the experiment (~19%). It should also be noted that cooperators, besides producing MVA, would also synthesize pantothenate. Since these constituted around 80% of the total population at the 72 h post-induction time point, it could be speculated that extracellular pantothenate concentrations would be high at this point. Therefore, an increasing number of cells could have exploited this freely available pantothenate in order to survive without the need to keep the pMTL71301::*araC*-PBAD-*mvaES*::*panC* plasmid. Consistently with these predictions, the levels of cheaters increased sharply (by 31.73%) between the 72 h and 96 h post-induction time points, while MVA productivity decreased from 0.044 to 0.028 g/L/h (-36.36%), during the same time interval. Even though a reduction in

the percentage of cheaters (-11.59%) was observed in the following time interval, between the 96 h and 120 h post-induction time points, MVA productivity (0.024 g/L/h) also decreased slightly during this time. This is likely to reflect the significant decrease in the total number of viable cells observed during the same time interval (Figure 2B), which may indicate the presence of nutrient limitations in the culture media. Indeed, MVA productivity kept decreasing up to 0.011 g/L/h between the 120 h and 144 h post-induction time points, even though the percentage of cheaters did not change significantly during this time. It is possible that MVA production may have stopped completely around the 144 h time point, when the highest MVA titre was recorded. Indeed, MVA concentration decreased slightly during the last 24 h time interval. Overall, our observations suggest that the implementation of the *panC*-based plasmid addiction system in *C. necator* H16 provided significant advantages in terms of MVA production, when applied to batch fermentation processes carried out under chemolithoautotrophic conditions. Approximately 4 g/L MVA were produced by the PAN strain, while no MVA synthesis was observed in the CTRL strain (no plasmid addiction system). However, the data obtained also show that the overproduction of pantothenate, resulting from the presence of multiple copies of the *panC* gene in each cell, led to the generation of significant levels of plasmid-free social cheaters. Although it would have been interesting to measure the variations in pantothenate levels over time to identify the concentration range allowing for high MVA productivity, these were not analysed since we considered this investigation to be beyond the scope of the present study.

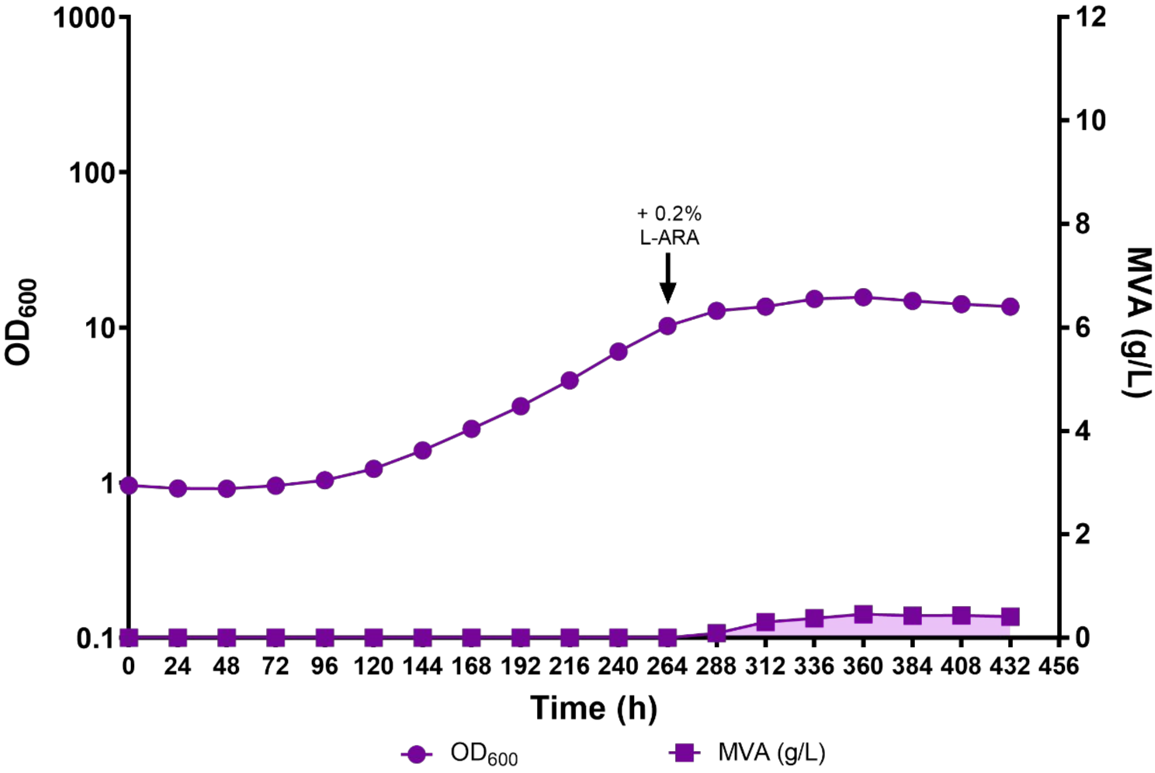

Figure S5. Growth curve and MVA production by ΔPHB_phaA.

Purple dots represent the average of 3 OD_{600} values measured at each time point, while purple squares correspond to the MVA titres produced at each time point by ΔPHB_phaA. MVA production is highlighted by the shaded area. The time of induction with 0.2% L-arabinose is indicated by the black arrow.

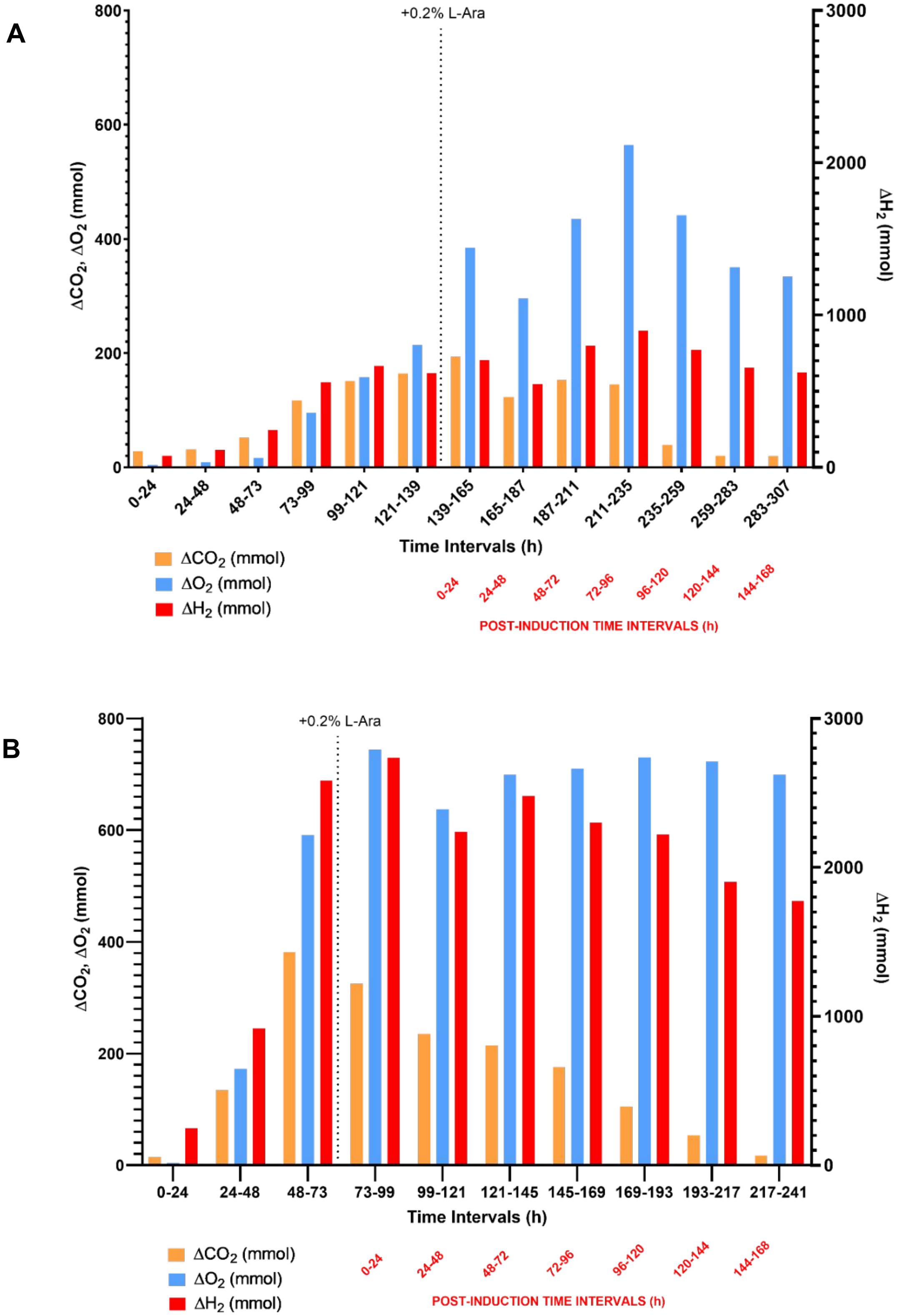

A
+0.2% L-Ara
ΔCO2, ΔO2 (mmol)
ΔH2 (mmol)
0-24
24-48
48-73
73-99
99-121
121-139
139-165
165-187
187-211
211-235
235-259
259-283
283-307
Time Intervals (h)
ΔCO2 (mmol)
ΔO2 (mmol)
ΔH2 (mmol)
0-24
24-48
48-72
72-96
96-120
120-144
144-168
POST-INDUCTION TIME INTERVALS (h)
B
+0.2% L-Ara
ΔCO2, ΔO2 (mmol)
ΔH2 (mmol)
0-24
24-48
48-73
73-99
99-121
121-145
145-169
169-193
193-217
217-241
Time Intervals (h)
ΔCO2 (mmol)
ΔO2 (mmol)
ΔH2 (mmol)
0-24
24-48
48-72
72-96
96-120
120-144
144-168
POST-INDUCTION TIME INTERVALS (h)

C

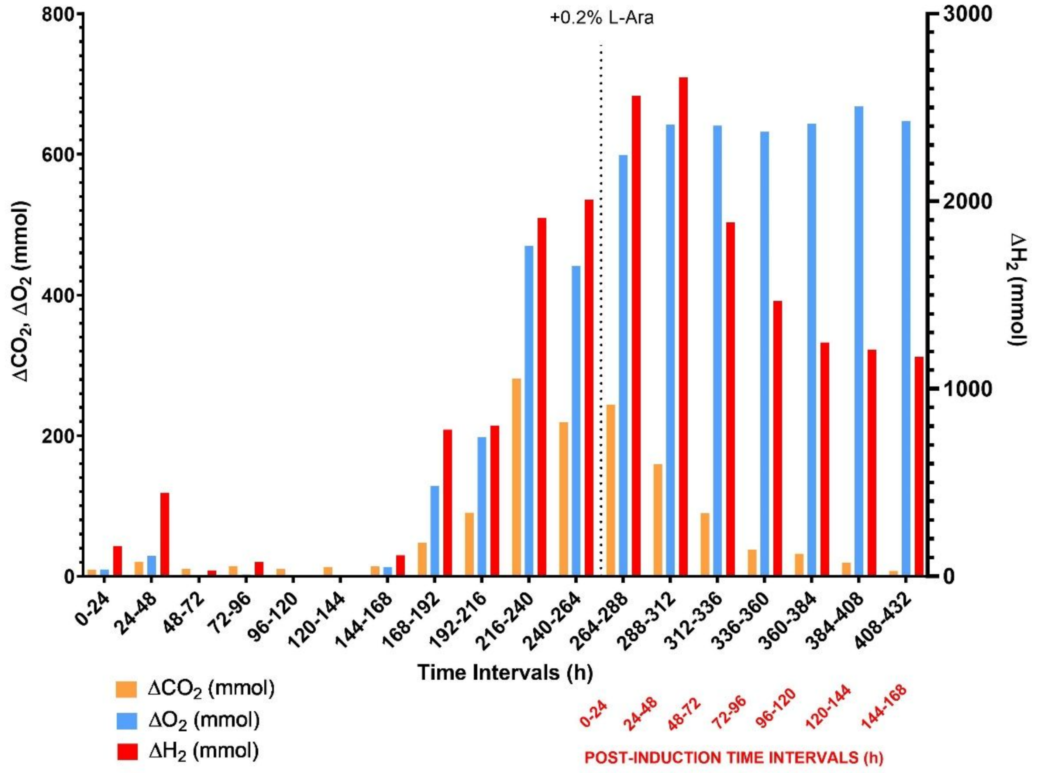

+0.2% L-Ara
ΔCO_2, ΔO_2 (mmol)
ΔH_2 (mmol)
Time Intervals (h)
0-24
24-48
48-72
72-96
96-120
120-144
144-168
168-192
192-216
216-240
240-264
264-288
288-312
312-336
336-360
360-384
384-408
408-432
ΔCO_2 (mmol)
ΔO_2 (mmol)
ΔH_2 (mmol)
POST-INDUCTION TIME INTERVALS (h)

D

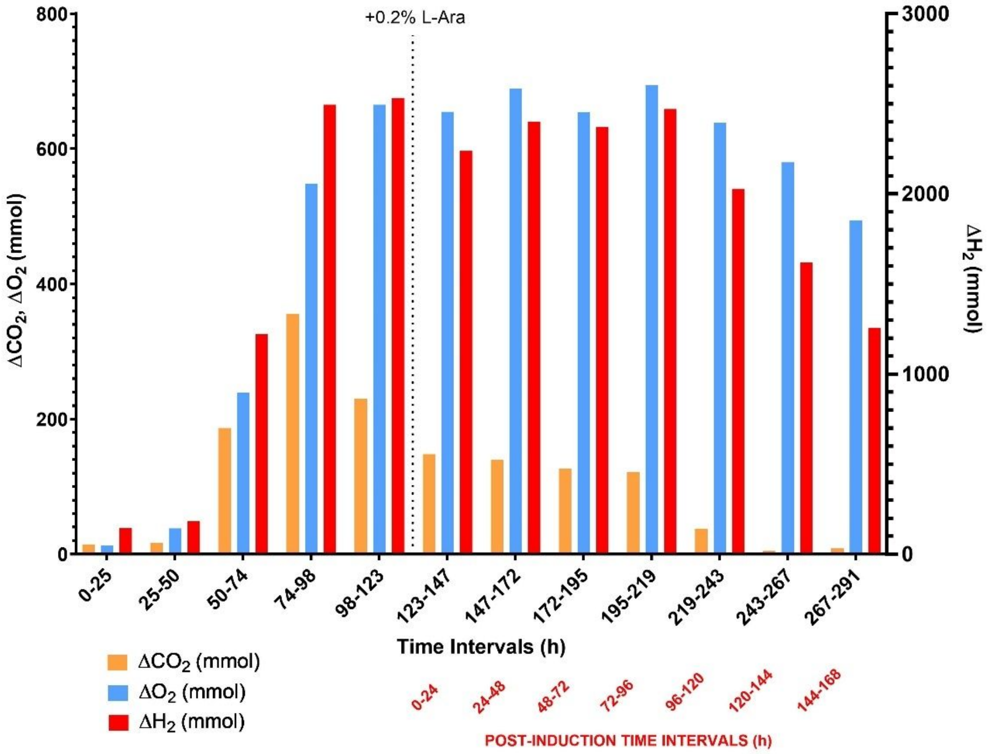

+0.2% L-Ara
ΔCO_2, ΔO_2 (mmol)
ΔH_2 (mmol)
Time Intervals (h)
0-25
25-50
50-74
74-98
98-123
123-147
147-172
172-195
195-219
219-243
243-267
267-291
ΔCO_2 (mmol)
ΔO_2 (mmol)
ΔH_2 (mmol)
POST-INDUCTION TIME INTERVALS (h)

E

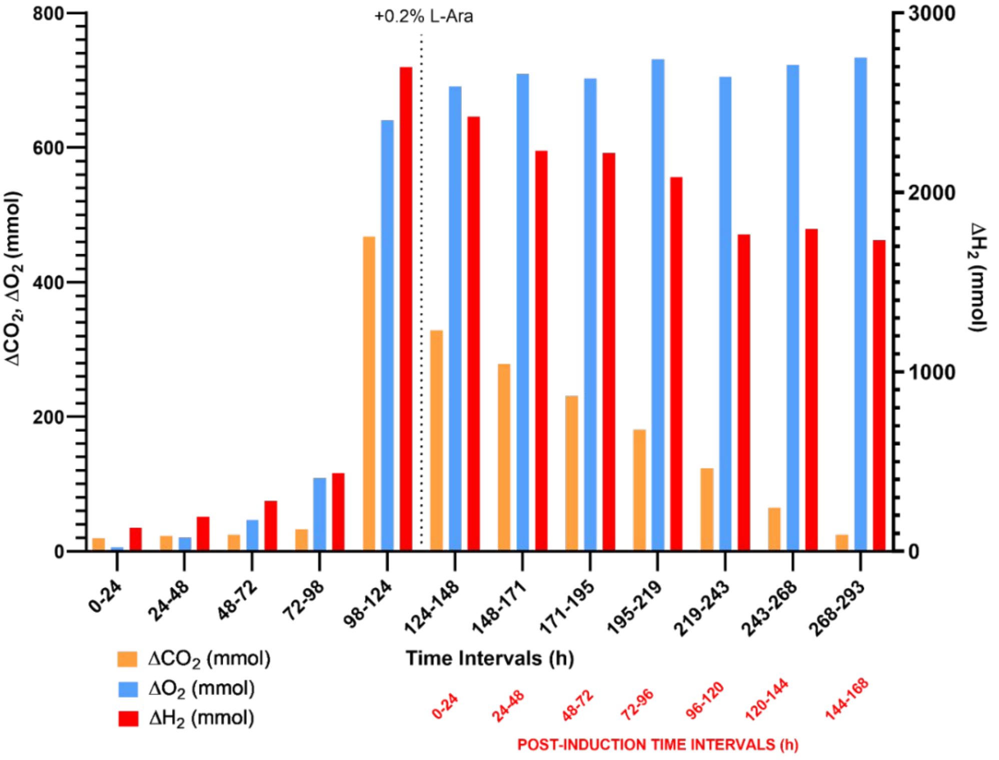

F

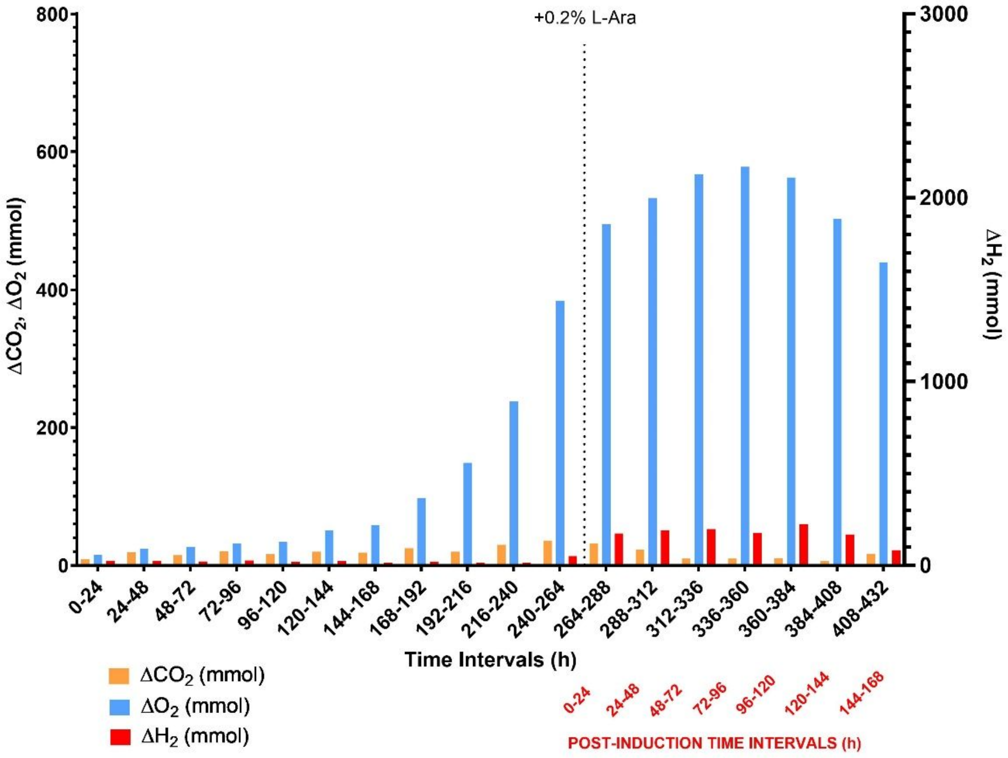

Figure S6. Total amount of gas consumed by CTRL, PAN, CBB, KI, CBB_phaA and ΔPHB_phaA per 24 h time interval.

B

Total amounts of each gas consumed by **A)** CTRL, **B)** PAN, **C)** CBB, **D)** KI, **E)** CBB_phaA and **F)** ΔPHB_phaA during the time intervals indicated on the horizontal axis of each graph. Orange bars = ΔCO_2; light blue bars = ΔO_2; red bars = ΔH_2. The dotted lines indicate the times of induction with 0.2% L-arabinose. Post-induction time intervals are highlighted in red.

Table S3. Amounts of CO_2, O_2 and H_2 gases consumed by CTRL, PAN, CBB, KI, CBB_phaA and ΔPHB_phaA after induction with 0.2% L-arabinose.

ΔCO_2, ΔO_2 and ΔH_2 respectively indicate the mmol of CO_2, O_2 and H_2 consumed during each of the post-induction time intervals by **A)** CTRL, **B)** PAN, **C)** CBB, **D)** KI, **E)** CBB_phaA and **F)** ΔPHB_phaA. ΔCO_2, ΔO_2 and ΔH_2 TOT are the total mmol of each gas consumed by each strain from the time of induction until the end of the fermentation processes.

A

		**Post-Induction Time intervals (h)**							
		0-24	24-48	48-72	72-96	96-120	120-144	144-168	**TOT**
**CTRL**	**ΔCO_2 (mmol)**	193.662	122.769	152.663	145.314	39.211	20.450	19.608	**693.677**
	**ΔO_2 (mmol)**	384.693	295.466	435.093	563.921	441.403	350.335	334.280	**2805.190**
	**ΔH_2 (mmol)**	703.399	545.177	798.792	897.695	771.545	654.293	622.684	**4993.585**

B

		**Post-Induction Time intervals (h)**							
		0-24	24-48	48-72	72-96	96-120	120-144	144-168	**TOT**

PAN									
	**ΔCO_2 (mmol)**	324.822	234.897	214.778	175.403	104.927	53.948	17.301	**1126.077**
	**ΔO_2 (mmol)**	744.190	637.274	699.181	710.161	730.193	722.632	699.363	**4942.994**
	**ΔH_2 (mmol)**	2734.743	2239.177	2478.869	2298.787	2221.346	1902.967	1772.504	**15648.393**

C

		Post-Induction Time intervals (h)							
		0-24	24-48	48-72	72-96	96-120	120-144	144-168	**TOT**
**CBB**	**ΔCO_2 (mmol)**	243.753	159.261	89.732	38.253	32.120	19.429	7.337	**589.886**
	**ΔO_2 (mmol)**	599.235	642.009	640.509	631.824	643.225	667.967	646.780	**4471.548**
	**ΔH_2 (mmol)**	2561.781	2659.268	1886.714	1467.985	1246.720	1210.014	1170.267	**12202.749**

D

		Post-Induction Time intervals (h)							
		0-24	24-48	48-72	72-96	96-120	120-144	144-168	**TOT**
**KI**	**ΔCO_2 (mmol)**	148.020	140.013	126.818	121.729	37.592	5.222	8.695	**588.089**
	**ΔO_2 (mmol)**	654.869	688.864	654.396	694.022	638.256	580.091	494.320	**4404.817**
	**ΔH_2 (mmol)**	2237.388	2400.802	2371.125	2470.762	2027.535	1618.233	1255.875	**14381.719**

E

		Post-Induction Time intervals (h)							
		0-24	24-48	48-72	72-96	96-120	120-144	144-168	**TOT**
**CBB_phaA**	**ΔCO_2 (mmol)**	328.286	277.911	230.789	181.250	123.133	64.539	24.621	**1230.529**

	ΔO_2 (mmol)	690.729	709.299	702.401	731.383	704.938	722.617	733.310	**4994.676**
	ΔH_2 (mmol)	2422.260	2232.550	2219.752	2084.310	1765.345	1795.083	1736.583	**14255.883**

F

		**Post-Induction Time intervals (h)**							
		0-24	24-48	48-72	72-96	96-120	120-144	144-168	**TOT**
**ΔPHB_ phaA**	ΔCO_2 (mmol)	31.599	22.570	9.785	9.982	10.369	6.376	16.751	**107.432**
	ΔO_2 (mmol)	494.765	532.454	567.533	577.912	562.707	502.676	439.589	**3677.636**
	ΔH_2 (mmol)	172.725	190.586	197.040	177.386	224.700	168.606	82.423	**1213.466**

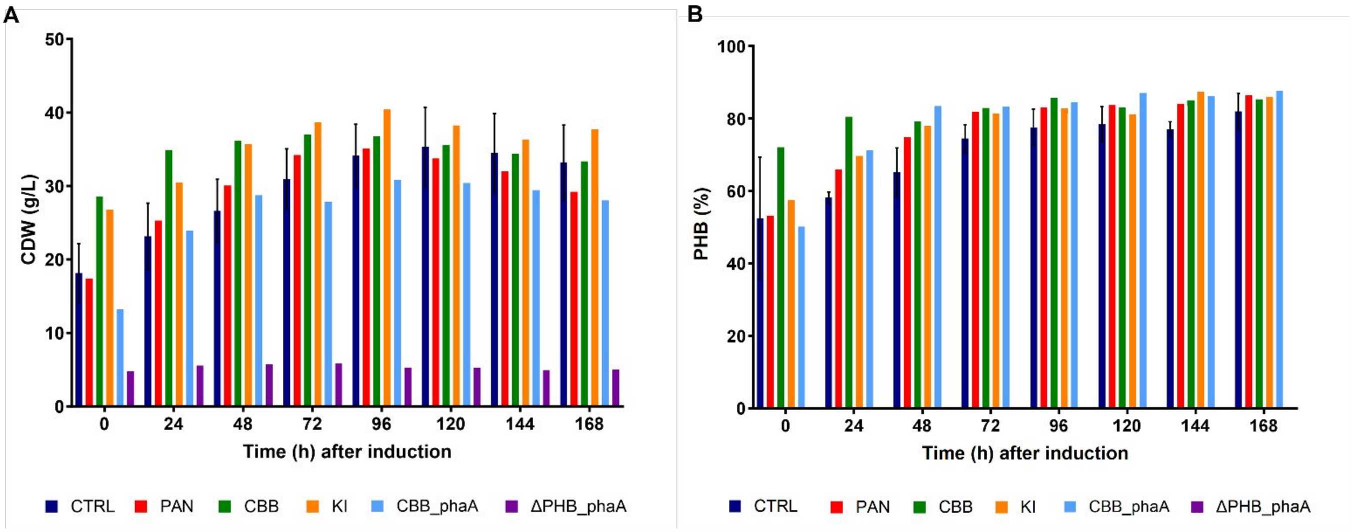

Figure S7. Determination of biomass production and PHB yields in strains CTRL, PAN, CBB, KI, CBB_phaA and ΔPHB_phaA, following induction.

A) Amounts of biomass (CDW, g/L) **B)** PHB yields, calculated as % of CDW, determined in strains CTRL, PAN, CBB, KI, CBB_phaA and ΔPHB_phaA at the time of induction (t=0 h) and every 24 h following this time point.

Table S4. MVA yields per mmol of CO_2 consumed by CTRL, PAN, CBB, KI, CBB_phaA and ΔPHB_phaA.

ΔCO_2 indicates the mmol of CO_2 consumed during each of the post-induction time intervals (as reported in Figure S6) by **A)** CTRL, **B)** PAN, **C)** CBB, **D)** KI, **E)** CBB_phaA and **F)** ΔPHB_phaA. ΔCO_2 TOT is the sum of these partial values, for each strain. ΔMVA represents the increments of MVA production (expressed in mmol) during each of the post-induction time intervals. These values were obtained by multiplying the MVA concentrations (mmol/L) detected at each time point by the volume of bacterial culture (in L) present in each bioreactor during the 24 h time interval preceding each culture sample collection time point. ΔMVA TOT is the total amount of MVA produced during each of the fermentation processes and was obtained by summing together all the partial ΔMVA amounts, for each strain. ΔMVA C-mmol represents the mmol of CO_2 required to produce the respective ΔMVA, during each time interval. These values were obtained by multiplying the partial ΔMVA amounts by 6 (ΔMVA (C-mmol) = ΔMVA (mmol) * 6). ΔMVA (C-mmol) TOT = ΔMVA TOT * 6. The partial and total MVA yields per mmol of CO_2 consumed were obtained using the following formula: MVA yield (% C-mol) = (ΔMVA (C-mmol) / ΔCO_2) * 100.

A

		Post-Induction Time intervals (h)							
		0-24	24-48	48-72	72-96	96-120	120-144	144-168	**TOT**
**CTRL**	**ΔCO_2 (mmol)**	193.662	122.769	152.663	145.314	39.211	20.450	19.608	**693.677**
	**ΔMVA (mmol)**	-	-	-	-	-	-	-	**-**
	**ΔMVA (C-mmol)**	-	-	-	-	-	-	-	**-**
	**MVA yield (% C-mol)**	-	-	-	-	-	-	-	**-**

B

		Post-Induction Time intervals (h)							
		0-24	24-48	48-72	72-96	96-120	120-144	144-168	**TOT**
**PAN**	**ΔCO_2 (mmol)**	324.822	234.897	214.778	175.403	104.927	53.948	17.301	**1126.077**
	**ΔMVA (mmol)**	3.992	3.542	5.327	3.258	2.889	1.268	-	**20.277**
	**ΔMVA (C-mmol)**	23.954	21.253	31.961	19.548	17.335	7.611	-	**121.663**
	**MVA yield (% C-mol)**	7.4	9.0	14.9	11.1	16.5	14.1	-	**10.8**

C

		Post-Induction Time intervals (h)							
		0-24	24-48	48-72	72-96	96-120	120-144	144-168	**TOT**
**CBB**	**ΔCO_2 (mmol)**	243.753	159.261	89.732	38.253	32.120	19.429	7.337	**589.886**
	**ΔMVA (mmol)**	6.491	7.855	5.805	1.831	2.020	0.138	0.685	**24.825**
	**ΔMVA (C-mmol)**	38.947	47.131	34.829	10.984	12.123	0.826	4.109	**148.948**

	MVA yield (% C-mol)	16.0	29.6	38.8	28.7	37.7	4.3	56.0	**25.3**

D

		Post-Induction Time intervals (h)							
		0-24	24-48	48-72	72-96	96-120	120-144	144-168	**TOT**
**KI**	**ΔCO_2 (mmol)**	148.020	140.013	126.818	121.729	37.592	5.222	8.695	**588.089**
	**ΔMVA (mmol)**	-	0.117	0.372	0.737	0.906	0.373	0.086	**2.591**
	**ΔMVA (C-mmol)**	-	0.700	2.233	4.421	5.439	2.236	0.519	**15.547**
	**MVA yield (% C-mol)**	-	0.5	1.8	3.6	14.5	42.8	6.0	**2.6**

E

		Post-Induction Time intervals (h)							
		0-24	24-48	48-72	72-96	96-120	120-144	144-168	**TOT**
**CBB_ phaA**	**ΔCO_2 (mmol)**	328.286	277.911	230.789	181.250	123.133	64.539	24.621	**1230.529**
	**ΔMVA (mmol)**	6.490	8.138	10.875	9.256	6.226	4.185	2.043	**47.213**
	**ΔMVA (C-mmol)**	38.942	48.827	65.248	55.534	37.355	25.110	12.260	**283.276**
	**MVA yield (% C-mol)**	11.9	17.6	28.3	30.6	30.3	38.9	49.8	**23.0**

F

		Post-Induction Time intervals (h)							
		0-24	24-48	48-72	72-96	96-120	120-144	144-168	**TOT**

ΔPHB_ phaA	**ΔCO_2 (mmol)**	31.599	22.570	9.785	9.982	10.369	6.376	16.751	**107.432**
	**ΔMVA (mmol)**	0.435	1.048	0.341	0.362	-	-	-	**2.186**
	**ΔMVA (C-mmol)**	2.610	6.290	2.048	2.169	-	-	-	**13.117**
	**MVA yield (% C-mol)**	8.3	27.9	20.9	21.7	-	-	-	**12.2**

Table S5. Comparison between the amounts of CO_2 (mM) consumed post-induction by strains CBB and CBB_phaA. Their respective titres (g/L) and yields (% C-mol or % CDW) for MVA and PHB (post-induction) are also reported. Standard deviation of <0.05 was observed between replicates (regression analysis).

Strain	CO_2 consumed P.I. (mM)	MVA produced (g/L)	MVA yield (% C-mol)	PHB produced P.I. (g/L)	PHB yield P.I. (% CDW)
CBB	589.9	5.2	25.0	10.4	13.2
CBB_phaA	1230.5	10.0	23.0	19.8	37.3

References:

Ehsaan, M., Baker, J., Kovacs, K., Malys, N., Minton, N. P., 2021. The pMTL70000 modular, plasmid vector series for strain engineering in Cupriavidus necator H16. J Microbiol Methods. 189, 106323. 10.1016/j.mimet.2021.106323.

Katsuki, H., Bloch, K., 1967. Studies on the biosynthesis of ergosterol in yeast. Formation of methylated intermediates. J Biol Chem. 242, 222-7.

Tabata, K., Hashimoto, S., 2004. Production of mevalonate by a metabolically-engineered Escherichia coli. Biotechnol Lett. 26, 1487-91. 10.1023/B:BILE.0000044449.08268.7d.

Wang, C., Yoon, S. H., Shah, A. A., Chung, Y. R., Kim, J. Y., Choi, E. S., Keasling, J. D., Kim, S. W., 2010. Farnesol production from Escherichia coli by harnessing the exogenous mevalonate pathway. Biotechnol Bioeng. 107, 421-9. 10.1002/bit.22831.

Yoon, S. H., Lee, S. H., Das, A., Ryu, H. K., Jang, H. J., Kim, J. Y., Oh, D. K., Keasling, J. D., Kim, S. W., 2009. Combinatorial expression of bacterial whole mevalonate pathway for the production of beta-carotene in E. coli. J Biotechnol. 140, 218-26. 10.1016/j.jbiotec.2009.01.008.

Zhu, W. L., Cui, J. Y., Cui, L. Y., Liang, W. F., Yang, S., Zhang, C., Xing, X. H., 2016. Bioconversion of methanol to value-added mevalonate by engineered Methylobacterium extorquens AM1 containing an optimized mevalonate pathway. Appl Microbiol Biotechnol. 100, 2171-82. 10.1007/s00253-015-7078-z.
